# Supplementary material for: Nanoparticles as an antidote for poisoned gold single-atom catalysts in sustainable propylene epoxidation
Source: Nat Commun. 2024 Apr 16;15:3249. doi: 10.1038/s41467-024-47538-4 (PMC11021464; doi:10.1038/s41467-024-47538-4)
Supplement: Supplementary file 1 — Supplementary Information [file 41467_2024_47538_MOESM1_ESM.pdf]

<u>Supplementary information</u>

Nanoparticles as an Antidote for Poisoned Gold Single-Atom Catalysts in Sustainable Propylene Epoxidation

Qianhong Wang,[‡,1] Keng Sang,[‡,1] Changwei Liu,[‡,1] Zhihua Zhang,[1] Wenyao Chen,[*,1] Te Ji,[2] Lina Li,[2] Cheng Lian,[1] Gang Qian,[1] Jing Zhang,[1] Xinggui Zhou,[*,1] Weikang Yuan,[1] Xuezhi Duan[*,1]

[1]State Key Laboratory of Chemical Engineering, East China University of Science and Technology, 130 Meilong Road, Shanghai 200237, China.

[2]Shanghai Synchrotron Radiation Facility, Shanghai Advanced Research Institute, Shanghai 201210, China.

‡ These authors contributed equally to this work

**Corresponding author: wenyao.chen@ecust.edu.cn; xgzhou@ecust.edu.cn; xzduan@ecust.edu.cn; Fax: +86-21-64253528.*

Model and simulation details

1. DFT calculations.

The adsorption and reaction of reactants and intermediates were calculated on a Au(111) surface that consists of five gold layers (45 atoms in total). The top three layers were relaxed, and the bottom two layers were fixed at the bulk lattice positions. A vacuum layer of 15 Å was set between the periodically repeated slabs to avoid inter-slab interactions. The adsorption and reaction of reactants and intermediates were also calculated on Au_{13} cluster and Au single atom over silicalite-1 surface. Firstly, we considered the orthorhombic cell of MFI for the calculations, as it is experimentally the most stable cell after thermal treatment. The bulk cell parameters and initial ionic positions were obtained from the International Zeolite Association database[1] and then reoptimized in the purely siliceous form. The final values are (almost orthorhombic: $\alpha = 90.00°$, $\beta = 90.00°$, $\gamma = 90.00°$): a = 20.282 Å, b = 19.938 Å, and c = 13.395 Å, in good agreement with the experimental values (orthorhombic: a = 20.07 ± 0.01 Å, b = 19.92 ± 0.01 Å and c = 13.42 ± 0.01 Å).[2,3] The silicic structure was cleaved along the (100) surface orientation, to mimic the relevant surface orientations identified experimentally, because the surface free energy of the (100) surface has been proved lower than that of (010) and (101) surfaces.[4] A 20 Å vacuum layer was added on top of the surfaces. The dimension of the cell was 19.937 × 13.396 × 44.018 Å for the (100) direction. Finally, the surface was saturated with OH groups, hydrogen atoms were added to monocoordinated O atoms for geometric optimization. After that, Au single atom and Au_{13} cluster models were constructed via an investigation of different local

structures of Au species on silicalite-1 surface in Supplementary Fig. 16, 17, respectively.

2. Catalytic performance evaluation.

The reported C_3H_6 conversions were determined based on the moles of carbon-containing products, as the measurements using the propylene peak often yielded values lower than 5%, which is in close proximity to the instrument's accuracy threshold. The C_3H_6 conversion, PO selectivity, PO formation rate, H_2 efficiency, and carbon balance were calculated as follows:

C_3H_6 conversion=moles of (C_3-oxygenates+2/3ethanal+1/3CO_2)/moles of propylene in the feed.

PO selectivity=moles of PO/moles of (C_3-oxygenates+2/3ethanal+1/3CO_2).

PO formation rate=(C_3H_6 mole flow rate×C_3H_6 conversion×PO selectivity)/(moles of Au×reaction time)

H_2 efficiency=moles of PO/moles of H_2 converted.

Carbon balance=moles of [2×ethanal+CO_2+3×(C_3-oxygenates+propylene)]/mole of 3×propylene in the feed.

It should be noted that the CO_2 can be well-analyzed in this work, and the carbon balance in all experiments was found to be better than 95%.

3. Hybrid classical MD technique and ReaxFF method.

Similarly, we utilized the silicalite-1 surface as the catalyst support for Au single

atoms and Au nanoparticles, which contains 1152 tetrahedrally coordinated Si atoms. The zeolite framework was electrically neutral and the monocoordinated O atoms were saturated with hydrogens. To investigate the mechanism of propylene poisoning on Au single atom, we initially constructed a system comprising only Au single atoms (Au_1). Specifically, 36 Au single atoms were equilibrated at the desired reactive temperature using the Berendsen thermostat.[5] The Au single atoms were then physisorbed onto the silicalite-1 surface using a Lennard-Jones potential as described previously. Following this, propylene (C_3H_6), hydrogen (H_2), and oxygen (O_2) molecules were randomly distributed around the catalysts. During the simulation, periodic boundary conditions were applied along the x, y, and z directions, and a constant volume and constant temperature (NVT) ensemble were employed. After performing structure optimization to achieve the minimum energy, we employed a rigid molecular zeolite structure to maintain the stability of the structure. A time step of 0.25 fs for 1×10^7 iterations (up to 2.0 ns) was assigned at 473 K after 50 ps of relaxation at 298.15 K via a Berendsen thermostat with a damping constant of 100 fs. For comparison, we introduced a 330-atom Au nanoparticle onto the zeolite surface, creating a system that consisted of both Au single atoms and Au nanoparticles (Au_{1+n}). Additionally, we constructed a system exclusively composed of gold nanoparticles (Au_n). A 0.3 bond order cutoff was used to recognize the molecules and analyze the species.

The system temperature was initially at 0 K and then increased rapidly at a rate of 20 K/ps until it reached 298.15 K. Following this, the system equilibrated at 298.15 K for a duration of 50 ps. In each simulation run, a Berendsen thermostat with a damping

constant of 100 fs was utilized to control the temperature. During the initial equilibrium relaxation stage, a time step of 0.25 fs was employed. Once the equilibrium was achieved, the temperature was raised from 298.15 K to the desired preset temperature of 473.15 K. This temperature increase was performed gradually with a heating rate of 35.6 K/ps. Subsequently, the system remained at this elevated temperature for a simulation period of 2 ns.

The reactants adsorption based on ReaxFF potential was also investigated in Supplementary Fig. 47, and a further comparison of the adsorption energy between ReaxFF potential and DFT calculations is made in Supplementary Fig. 48. It can be seen that both methods demonstrate consistent trends in the adsorption capabilities of various species on the Au (111) surface and Au single atom.

4. Ratio of Au SAs and Au NPs for the mortar-mixing catalyst (Au_n:Au_1=1:7).

For the mortar-mixing catalyst (Au_n:Au_1=1:7) with the amount of 0.15 g, the weight for Au_n and Au_1 is determined to be 0.01875 g and 0.13125 g, respectively.

For the Au_n catalyst with the loading of 0.026 wt%, the molar amount of Au atom (n_{Au}) is determined to be:

$n_{Au}=0.01875*0.026/100/195=2.5*10^{-8}$ mol

Moreover, for Au_n catalyst with Au nanoparticle size of 2.1 nm, the number of Au atom for each nanoparticle can be estimated based on our previous study as 286.[6] Hence, the molar of Au NPs is determined to be:

$n_{NPs}=n_{Au}/286=8.74*10^{-11}$ mol

For the Au_1 catalyst with the loading of 0.004 wt%, the molar amount of Au atom ($n_{Au'}$) is determined to be:

$n_{Au'}$=0.13125*0.004/100/195=2.69*10^{-8} mol

Hence, the molar of Au SAs is determined to be:

n_{SAs}=$n_{Au'}$=2.69*10^{-8} mol

In this regard, the molar ratio of Au NPs to Au SAs (n_{NPs}:n_{SAs}) is calculated as 1:308.

Supplementary Table S1. The nominal and actual Au loadings as well as particle size of Au/S-1 catalysts.

Sample	Nominal loading (wt%)	Actual loading (wt%)	Particle size
1	0.04	0.004	-
2	0.05	0.014	-
3	0.06	0.026	2.1
4	0.08	0.051	2.3
5	0.10	0.070	2.5
6	0.20	0.149	2.8
7	0.30	0.230	3.2
8	0.60	0.461	3.9

Supplementary Table S2. The catalytic performances of Au/S-1 catalysts with different loadings.

No.	Loading (wt%)	Selectivity (%)						PO formation rate ($mol_{PO}\cdot mol_{Au}^{-1}\cdot h^{-1}$)	H_2 efficiency
		PO	Ethanal	Acrolein	Propanal	Acetone	CO_2		
1	0.004	25.1	9.5	7.9	49.7	6.6	1.2	10.4	1.4%
2	0.014	55.2	9.2	13.7	6.0	2.4	13.6	80.2	13.6%
3	0.026	26.7	14.6	21.0	11.9	6.1	19.6	25.9	2.6%
4	0.051	19.0	14.5	20.2	13.0	6.1	27.3	11.8	1.1%
5	0.070	11.8	11.1	27.7	11.6	7.0	30.9	4.6	0.3%
6	0.149	3.0	11.2	32.5	13.1	9.4	30.8	1.1	0.2%
7	0.230	1.5	9.4	38.3	11.7	8.4	30.7	0.5	0.1%
8	0.461	0.4	7.3	47.2	8.0	7.7	29.3	0.1	0.1%

Supplementary Table S3. Parameters used for eliminating the internal and external transfer limitations.

Parameter	Description	Value	Unit
$-r_{obs}$	Max observed PO rate	3.86×10^{-5}	$mol/(kg_{cat}\cdot s)$
ρ_c	Solid density of catalyst	350	kg/m^3
R	Particle size (200 mesh)	7.5×10^{-5}	m
C_{AS}	Maximum surface concentration of C_3H_6	5.12	mol/m^3
De	Effective diffusity (calculated by double molecular diffusion model	2.92×10^{-6}	m^2/s
D_{AB}	Gas-phase diffusivity	2.74×10^{-5}	m^2/s
ε_P	Pellet porosity	0.4	/
σ_C	Constriction factor	0.8	/
τ	Tortuosity	3	/
n	Maximum reaction order	1	/
C_{Ab}	Maximum C_3H_6 bulk concentration	5.12	mol/m^3
kc	Mass transfer coefficient	0.089	m/s

External Diffusion: Mears Criteria

The absence of external mass transfer limitations can be evaluated using the Mears criteria:

$$M = \frac{-r_{obs} \cdot \rho_c \cdot R \cdot n}{k_c \cdot C_{Ab}} < 0.15 \qquad (1)$$

k_c was calculated from the Sherwood number using the following equation:

$$\frac{k_c d_P}{D_A} = 0.91 \cdot Re^{0.49} \cdot Sc^{1/3} \tag{2}$$

Putting the values of Table S3 in Eq. (1), we can get:

$$M_{\max} = \frac{-r_{obs} \cdot \rho_c \cdot R \cdot n}{k_c \cdot C_{Ab}} = 2.22 \times 10^{-6} << 0.15 \tag{3}$$

Hence, the external mass transfer limitations during kinetics experiment can be eliminated.

Internal diffusion: Weisz-Prater Criteria

The effect of internal mass transfer limitations was evaluated using the Weisz-Prater criteria:

$$WP = \frac{-r_{obs} \cdot \rho_c \cdot R^2}{De \cdot C_{AS}} < 1 \tag{4}$$

The De can be calculated as:

$$De = \frac{D_{AB} \cdot \varepsilon_P \cdot \sigma_c}{\tau} \tag{5}$$

D_{AB} for a mixture of C_3H_6-N_2 was calculated by Chapman-Enskog Equation:

$$D_{AB} = \frac{0.00143 T^{1.75}}{P M_{AB}^{0.5} [(\sum v)_A^{1/3} + (\sum v)_B^{1/3}]^2} \tag{6}$$

Putting the values of Table S3 in Eq. (4), we can get:

$$WP_{\max} = \frac{-r_{obs} \cdot \rho_c \cdot R^2}{De \cdot C_{AS}} = 5.08 \times 10^{-6} << 1 \tag{7}$$

Thus, this reaction system during kinetics experiment does not suffer from external and internal mass transfer limitation.

Supplementary Table S4. The catalytic performances for the Au_1 and Au_n catalyst in different intimacies at a given proportion of Au_n to Au_1 (mass ratio of 1:3).

Sample	PO formation rate ($mol_{PO}\cdot h^{-1}\cdot mol_{Au}^{-1}$)	PO selectivity (%)	H_2 efficiency (%)
Au_n	25.9	26.7	2.6
Au_1 up || Au_n down	59.5	29.3	5.4
Mortar mixing	170.1	58.1	10.6
Au_n up || Au_1 down	60.0	38.8	7.1
Au_1	10.4	25.1	1.4

Supplementary Table S5. The catalytic performances for the mortar mixing Au_1 and Au_n catalyst in different proportions.

Au_1:Au_n	PO formation rate ($mol_{PO} \cdot h^{-1} \cdot mol_{Au}^{-1}$)	PO selectivity (%)	H_2 efficiency (%)
0:1	25.9	26.7	2.6
1:3	48.1	37.4	4.9
1:1	69.5	41.4	7.3
3:1	170.1	58.1	10.6
5:1	258.4	59.1	14.2
7:1	583.6	75.6	30.5
9:1	129.5	51.7	16.2
1:0	10.4	25.1	1.4

Supplementary Table S6. The comparison of PO formation rate between the monometallic Au catalyst in the current study and bifunctional Au-Ti catalysts in previous studies.

Sample	PO formation rate ($mol_{PO}\cdot h^{-1}\cdot mol_{Au}^{-1}$)	PO selectivity	H_2 efficiency	C_3H_6 conversion	Reference
Au/U-TS-1	2034.9	92.4	55	2.0	*J. Catal.* 2014, 313, 104–112
Au/TS-1	1800.0				*J. Catal.* 2013, 308, 98–113
Au/TS-1	1695.7				*J. Catal.* 2012, 287, 178–189
Au/TS-1-B	994.8	95	30.9		*Appl. Catal. B* 2022, 319, 121837
Au/TS-1-B-DPA	847.8	89.6	40	3.2	*J. Catal.* 2022, 416, 410–422
Au/TS-1	695.2	83	26		*ACS Catal*. 2018, 8, 10649–10657
**Au_1&Au_n**	**583.6**	**75.6**	**30.5**	**0.29**	***This work***
Au/TS-1	561.0	76	30	10	*J. Catal.* 2005, 232, 38–42
Au/TS-1	552.8		< 30		*J. Catal.* 2015, 325, 128–135
Au/TS-1-B	540.0	89.6	33.6		*J. Catal.* 2014, 317, 99–104
Au/HTS-1(NIMG)	509.7		29.1		*ACS Sustainable Chem. Eng*. 2022, 10, 9515–9524
Au/TS-1-B	508.7	92	35		*Ind. Eng. Chem. Res*. 2019, 58, 17300–17307
Au/S-1/TS-1@dendritic-SiO_2	486.3	93.9	26.1		*Green Energy Environ*. 2020, 5, 473–483
Au/STS-1	478.2	91.2			*Catal. Today*. 2020, 347, 102–109
Au/TS-1-cS	474.8	90.7	43.6		*Engineering* 2023, DOI: 10.1016/j.eng.2023.01.008
Au/TS-2-B	444.6	90	35		*AIChE J* 2020, 66, e16815
Au/HTS-1	423.9	85	~20		*ACS Appl. Mater. Interfaces* 2021, 13, 26134–26142
Au/TS-1-B	412.2	83			*Chem. Eng. J.* 2015, 278, 234–239
Au/Ti-SiO_2	410.4	91.9	14	4.8	*J. Catal.* 2016, 344, 434–444
Au/TS-1	387.1				*J. Catal.* 2018, 365, 105–114
Au/Ti-SiO_2	376.8	90	14.5		*J. Catal.* 2016, 338, 284–294
Au/MTS-1	370.4	95.2			*ACS Catal*. 2017, 7, 2668–2675

Au/TS-1-B	353.3	83	22		*Appl. Catal. B* 2014, 150–151, 396–401
Au/TS-1-B	352.7	85	22	4.3	*ACS Catal*. 2018, 8, 7799–7808
Au/TS-1(SG)	215.4	84	24	8.3	*J. Catal.* 2011, 278, 8–15
Au/S-1/TS-1	195.9				*J. Catal.* 2012, 296, 31–42
Au/Ge-TS-1	159.9	91		4	*J. Catal.* 2009, 267, 202–206
Au-Ti@MFI	139.0	83.9	23	14.8	*J. Mater. Chem. A* 2020, 8, 4428–4436
Au/Mg-TS-1	119.4	85.8		5.7	*Catal. Today* 2009,147, 186–195
Au/TS-1	81.5	72.1	27.6	10.1	*J. Catal.* 2011, 283, 192–201
Au/TiO_2@SBA-15	70.5	62	7.5	2.2	*J. Catal.* 2011, 282, 94–102
Au/TS-1	27.4	77.2		1.1	*J Solid State Chem.* 2018, 261, 92–102
Au-PVP/TS-1	21.6				*ACS Catal.* 2022, 12, 16, 10147–10160
Au/TS-1(PT)	7.6	88	23	1.3	*Angew. Chem. Int. Ed.* 2021, 133, 18333–18341

Supplementary Table S7. The comparison of reaction orders of H_2, O_2 and propylene for the mortar mixing Au_1 and Au_n catalyst in different ratios.

Au_1:Au_n	Reaction order			n_{C3H6}-n_{H2}
	n_{H2}	n_{O2}	n_{C3H6}	
0:1	0.19	0.22	0.47	0.28
1:3	0.21	0.23	0.42	0.21
1:1	0.24	0.25	0.40	0.16
3:1	0.28	0.31	0.35	0.07
5:1	0.32	0.28	0.33	0.01
7:1	0.45	0.29	0.32	-0.13
9:1	0.61	0.34	0.26	-0.35
1:0	0.73	0.35	0.20	-0.53

Supplementary Table S8. Atomic coordinates of the optimized model in Supplementary Fig. S18.

Atom	X	Y	Z	Atom	X	Y	Z	Atom	X	Y	Z
O1	0.0345	0.8472	0.2007	O41	0.1096	0.4334	0.0863	O81	0.3228	0.9935	0.2966
O2	0.0339	0.9369	0.1475	O42	0.1308	0.6479	0.1091	O82	0.5952	0.9236	0.4294
O3	0.1020	0.7695	0.1549	O43	0.0961	0.5734	0.1622	O83	0.6252	0.8433	0.3769
O4	0.9681	0.7657	0.1544	O44	0.1251	0.3862	0.1438	O84	0.5981	0.6864	0.3439
O5	0.0062	0.1056	0.1188	O45	0.5345	0.4349	0.1978	O85	0.6256	0.8600	0.3165
O6	0.9165	0.9576	0.1211	O46	0.5252	0.3438	0.2505	O86	0.6277	0.6359	0.2879
O7	0.0259	0.9415	0.0871	O47	0.6013	0.5041	0.2449	O87	0.5965	0.6977	0.2325
O8	0.0936	0.2439	0.1030	O48	0.4680	0.5215	0.2434	O88	0.6256	0.8752	0.2563
O9	0.0078	0.1701	0.0617	O49	0.5015	0.1785	0.2804	O89	0.8924	0.4349	0.1978
O10	0.9650	0.2884	0.1061	O50	0.4145	0.3323	0.2834	O90	0.9014	0.3452	0.2509
O11	0.0899	0.2340	0.0183	O51	0.5308	0.3434	0.3110	O91	0.8250	0.5040	0.2446
O12	0.0065	0.0846	0.0074	O52	0.5868	0.0305	0.2899	O92	0.9585	0.5226	0.2431
O13	0.0401	0.9048	0.0287	O53	0.5241	0.1176	0.3363	O93	0.9253	0.1797	0.2803
O14	0.9219	0.9315	0.0025	O54	0.4567	0.0019	0.2973	O94	0.0126	0.3329	0.2833
O15	0.0951	0.7858	0.0689	O55	0.6009	0.0377	0.3789	O95	0.8973	0.3438	0.3118
O16	0.8154	0.8567	0.1968	O56	0.5293	0.1958	0.3920	O96	0.8400	0.0312	0.2890
O17	0.7946	0.9218	0.1414	O57	0.5419	0.3797	0.3698	O97	0.9021	0.1166	0.3357
O18	0.8222	0.0960	0.1156	O58	0.4201	0.3019	0.3805	O98	0.9702	0.0027	0.2966
O19	0.8173	0.9334	0.0818	O59	0.6017	0.4955	0.3298	O99	0.8261	0.0372	0.3787
O20	0.7961	0.1479	0.0590	O60	0.3154	0.4254	0.2017	O100	0.8962	0.1968	0.3911
O21	0.8308	0.0734	0.0059	O61	0.2973	0.3520	0.2563	O101	0.8856	0.3810	0.3707
O22	0.8018	0.8862	0.0243	O62	0.3249	0.1876	0.2877	O102	0.0068	0.3033	0.3813
O23	0.9241	0.3484	0.4280	O63	0.3044	0.3569	0.3167	O103	0.8262	0.4958	0.3300
O24	0.8930	0.4369	0.0206	O64	0.3151	0.1161	0.3440	O104	0.1115	0.4254	0.2017
O25	0.8249	0.2695	0.0132	O65	0.3054	0.2120	0.3962	O105	0.1301	0.3518	0.2563
O26	0.9588	0.2657	0.0137	O66	0.3035	0.3978	0.3753	O106	0.1022	0.1881	0.2878
O27	0.9207	0.6056	0.0494	O67	0.4232	0.9351	0.4329	O107	0.1225	0.3576	0.3167
O28	0.0104	0.4576	0.0470	O68	0.4006	0.8529	0.3780	O108	0.1124	0.1166	0.3441
O29	0.9010	0.4415	0.0811	O69	0.3364	0.0177	0.3956	O109	0.1228	0.2126	0.3962
O30	0.8333	0.7439	0.0651	O70	0.4665	0.0238	0.3818	O110	0.1231	0.3992	0.3753
O31	0.9191	0.6701	0.1064	O71	0.4248	0.6818	0.3490	O111	0.0027	0.9365	0.4326
O32	0.9619	0.7884	0.0620	O72	0.5110	0.8347	0.3454	O112	0.0270	0.8549	0.3777
O33	0.8370	0.7340	0.1498	O73	0.3934	0.8415	0.3174	O113	0.0909	0.0189	0.3959
O34	0.9204	0.5846	0.1607	O74	0.3382	0.5399	0.3353	O114	0.9610	0.0261	0.3813
O35	0.8868	0.4048	0.1394	O75	0.4192	0.6137	0.2923	O115	0.0028	0.6830	0.3490
O36	0.0050	0.4315	0.1656	O76	0.4682	0.5002	0.3361	O116	0.9161	0.8359	0.3456
O37	0.8318	0.2858	0.0992	O77	0.3362	0.5420	0.2503	O117	0.0330	0.8423	0.3172
O38	0.0706	0.3588	0.4301	O78	0.4129	0.6984	0.2383	O118	0.0893	0.5413	0.3351
O39	0.1323	0.4218	0.0267	O79	0.3902	0.8829	0.2588	O119	0.0070	0.6130	0.2924
O40	0.1048	0.5960	0.0525	O80	0.5056	0.8414	0.2328	O120	0.9595	0.5005	0.3369

Supplementary Table S8. Atomic coordinates of the optimized model in Supplementary Fig. S18.

Atom	X	Y	Z	Atom	X	Y	Z	Atom	X	Y	Z
O121	0.0903	0.5415	0.2504	O161	0.5259	0.4415	0.0811	Si201	0.7908	0.1833	0.0942
O122	0.0145	0.6985	0.2385	O162	0.5936	0.7439	0.0651	Si202	0.7912	0.1639	0.0226
O123	0.0371	0.8835	0.2586	O163	0.5078	0.6701	0.1064	Si203	0.8423	0.9547	0.0000
O124	0.9213	0.8407	0.2329	O164	0.4650	0.7884	0.0620	Si204	0.7919	0.8464	0.0589
O125	0.1041	0.9939	0.2967	O165	0.5899	0.7340	0.1498	Si205	0.8927	0.3287	0.0037
O126	0.8319	0.9243	0.4293	O166	0.5065	0.5846	0.1607	Si206	0.9313	0.4857	0.0496
O127	0.8014	0.8430	0.3769	O167	0.5401	0.4048	0.1394	Si207	0.9087	0.7021	0.0710
O128	0.8291	0.6875	0.3435	O168	0.4219	0.4315	0.1656	Si208	0.9110	0.6888	0.1427
O129	0.8019	0.8618	0.3166	O169	0.5951	0.2858	0.0992	Si209	0.9267	0.4650	0.1658
O130	0.7992	0.6351	0.2878	O170	0.3515	0.3610	0.4304	Si210	0.8966	0.3540	0.1064
O131	0.8299	0.6974	0.2323	O171	0.2946	0.4218	0.0267	Si211	0.1363	0.3239	0.0050
O132	0.8010	0.8743	0.2563	O172	0.3222	0.5960	0.0525	Si212	0.0894	0.4770	0.0531
O133	0.3924	0.8472	0.2007	O173	0.3173	0.4334	0.0863	Si213	0.1361	0.6833	0.0739
O134	0.3930	0.9369	0.1475	O174	0.2961	0.6479	0.1091	Si214	0.1357	0.6639	0.1455
O135	0.3249	0.7695	0.1549	O175	0.3308	0.5734	0.1622	Si215	0.0846	0.4547	0.1681
O136	0.4588	0.7657	0.1544	O176	0.3018	0.3862	0.1438	Si216	0.1350	0.3464	0.1092
O137	0.4207	0.1056	0.1188	O177	0.7135	0.7846	0.1643	Si217	0.5316	0.4520	0.2341
O138	0.5104	0.9576	0.1211	O178	0.7135	0.2036	0.1035	Si218	0.4927	0.2986	0.2814
O139	0.4010	0.9415	0.0871	O179	0.7135	0.1639	0.0117	Si219	0.5173	0.0812	0.3012
O140	0.3333	0.2439	0.1030	O180	0.7135	0.8218	0.0647	Si220	0.5295	0.0930	0.3723
O141	0.4191	0.1701	0.0617	O181	0.2135	0.2846	0.0039	Si221	0.4980	0.3076	0.3921
O142	0.4619	0.2884	0.1061	O182	0.2135	0.7036	0.0646	Si222	0.5349	0.4306	0.3364
O143	0.3370	0.2340	0.0183	O183	0.2135	0.6639	0.1565	Si223	0.2909	0.4526	0.2358
O144	0.4204	0.0846	0.0074	O184	0.2135	0.3218	0.1034	Si224	0.3351	0.3075	0.2861
O145	0.3868	0.9048	0.0287	O185	0.2134	0.4902	0.2354	Si225	0.2938	0.0991	0.3087
O146	0.5050	0.9315	0.0025	O186	0.2135	0.0978	0.3052	Si226	0.2927	0.1055	0.3796
O147	0.3318	0.7858	0.0689	O187	0.2138	0.0756	0.3822	Si227	0.3455	0.3181	0.3958
O148	0.6115	0.8567	0.1968	O188	0.2136	0.4819	0.3393	Si228	0.2906	0.4437	0.3418
O149	0.6323	0.9218	0.1414	O189	0.7135	0.9681	0.4030	Si229	0.4071	0.9576	0.3972
O150	0.6048	0.0960	0.1156	O190	0.7138	0.5957	0.3327	Si230	0.4322	0.8029	0.3476
O151	0.6096	0.9334	0.0818	O191	0.7132	0.6087	0.2421	Si231	0.4121	0.5836	0.3279
O152	0.6308	0.1479	0.0590	O192	0.7135	0.9764	0.2888	Si232	0.4089	0.5932	0.2561
O153	0.5961	0.0734	0.0059	Si193	0.0342	0.8287	0.1644	Si233	0.4258	0.8164	0.2328
O154	0.6251	0.8862	0.0243	Si194	0.9956	0.9857	0.1185	Si234	0.3916	0.9310	0.2925
O155	0.5009	0.3526	0.4269	Si195	0.0183	0.2021	0.0971	Si235	0.6345	0.9441	0.3970
O156	0.5339	0.4369	0.0206	Si196	0.0159	0.1888	0.0254	Si236	0.5901	0.8070	0.3454
O157	0.6020	0.2695	0.0132	Si197	0.0002	0.9650	0.0023	Si237	0.6358	0.6027	0.3233
O158	0.4681	0.2657	0.0137	Si198	0.0303	0.8540	0.0617	Si238	0.6350	0.6116	0.2519
O159	0.5062	0.6056	0.0494	Si199	0.7906	0.8239	0.1632	Si239	0.5849	0.8179	0.2297
O160	0.4165	0.4576	0.0470	Si200	0.8375	0.9770	0.1150	Si240	0.6369	0.9354	0.2879

Supplementary Table S8. Atomic coordinates of the optimized model in Supplementary Fig. S18.

Atom	X	Y	Z	Atom	X	Y	Z
Si241	0.8951	0.4525	0.2341	Si281	0.5002	0.4650	0.1658
Si242	0.9342	0.2998	0.2818	Si282	0.5303	0.3540	0.1064
Si243	0.9095	0.0816	0.3006	Si283	0.2906	0.3239	0.0050
Si244	0.8970	0.0937	0.3718	Si284	0.3375	0.4770	0.0531
Si245	0.9285	0.3081	0.3932	Si285	0.2908	0.6833	0.0739
Si246	0.8929	0.4308	0.3374	Si286	0.2912	0.6639	0.1455
Si247	0.1359	0.4526	0.2358	Si287	0.3423	0.4547	0.1681
Si248	0.0920	0.3079	0.2861	Si288	0.2919	0.3464	0.1092
Si249	0.1332	0.0994	0.3087	H289	0.9755	0.3608	0.4370
Si250	0.1350	0.1054	0.3796	H290	0.3775	0.9487	0.4467
Si251	0.0819	0.3176	0.3953	H291	0.6241	0.9574	0.4483
Si252	0.1363	0.4449	0.3416	H292	0.0479	0.9510	0.4466
Si253	0.0198	0.9592	0.3970	H293	0.8028	0.9580	0.4482
Si254	0.9949	0.8037	0.3476	H294	0.4491	0.3648	0.4354
Si255	0.0147	0.5844	0.3282	H295	0.0310	0.9381	0.9713
Si256	0.0178	0.5932	0.2563	H296	0.8182	0.9286	0.9677
Si257	0.0012	0.8164	0.2329	H297	0.8935	0.3444	0.9691
Si258	0.0352	0.9316	0.2923	H298	0.1129	0.3513	0.9727
Si259	0.7926	0.9441	0.3969	H299	0.3959	0.9381	0.9713
Si260	0.8371	0.8076	0.3454	H300	0.6087	0.9286	0.9677
Si261	0.7921	0.6027	0.3233	H301	0.5334	0.3444	0.9691
Si262	0.7916	0.6114	0.2518	H302	0.3141	0.3513	0.9727
Si263	0.8420	0.8175	0.2297	H303	0.1200	0.3763	0.4405
Si264	0.7902	0.9358	0.2877	H304	0.3006	0.3751	0.4395
Si265	0.3927	0.8287	0.1644	Au305	0.7231	0.5609	0.4560
Si266	0.4313	0.9857	0.1185	Au306	0.8670	0.5468	0.4591
Si267	0.4087	0.2021	0.0971	Au307	0.7859	0.4967	0.5094
Si268	0.4110	0.1888	0.0254	Au308	0.7817	0.3687	0.4574
Si269	0.4267	0.9650	0.0023	Au309	0.5787	0.5785	0.4532
Si270	0.3966	0.8540	0.0617	Au310	0.6609	0.6227	0.4032
Si271	0.6363	0.8239	0.1632	Au311	0.7221	0.4328	0.4026
Si272	0.5894	0.9770	0.1150	Au312	0.6428	0.3890	0.4544
Si273	0.6361	0.1833	0.0942	Au313	0.6619	0.7521	0.4549
Si274	0.6357	0.1639	0.0226	Au314	0.7257	0.6893	0.5082
Si275	0.5846	0.9547	0.0000	Au315	0.6435	0.5169	0.5072
Si276	0.6350	0.8464	0.0589	Au316	0.8020	0.7342	0.4554
Si277	0.5342	0.3287	0.0037	Au317	0.8047	0.6018	0.4051
Si278	0.4956	0.4857	0.0496				
Si279	0.5183	0.7021	0.0710				
Si280	0.5159	0.6888	0.1427				

Supplementary Table S9. Lennard−Jones 12-6 parameters for Au species, S-1, and gas molecules.

Pair*	ε (Kcal/mol)	σ (Å)
2-1	1.523496	2.748392
2-4	0.093081	2.654878
2-5	0.093081	2.654878
2-6	0.183695	3.238417
2-7	0.094297	3.367597
2-8	0.228000	2.859785
3-1	0.638269	3.345217
3-4	0.038996	3.251703
3-5	0.038996	3.251703
3-6	0.076959	3.835241
3-7	0.039506	3.964422
3-8	0.095521	3.456609
4-1	0.621965	2.543486
4-4	0.038000	2.449971
4-5	0.038000	2.449971
4-6	0.074993	3.033510
4-7	0.038497	3.162690
4-8	0.093081	2.654878

*1~8 correspond to different atom types, see Supplementary Fig. 46 for details.

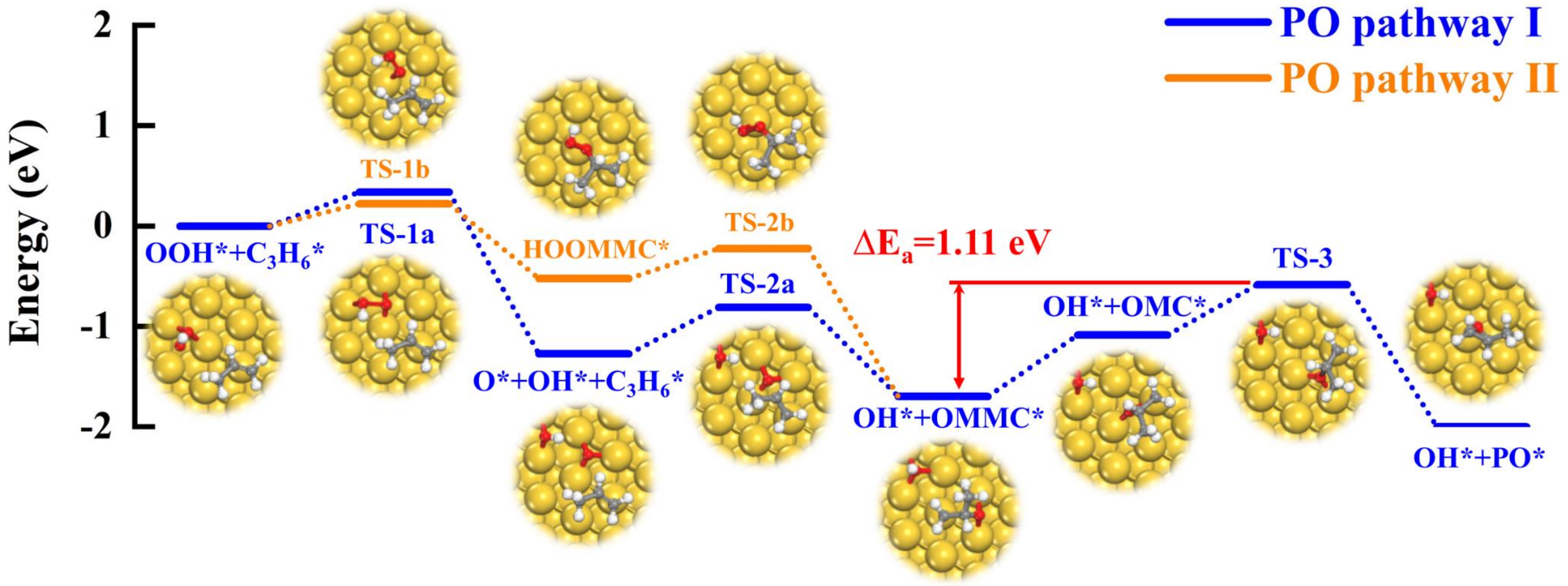

Supplementary Fig. 1. DFT calculations for PO formation. Calculated energy profile and the corresponding structural configurations for PO formation on Au(111) surface, which involves the attack of C_3H_6* by OOH* to generate PO* and OH*.

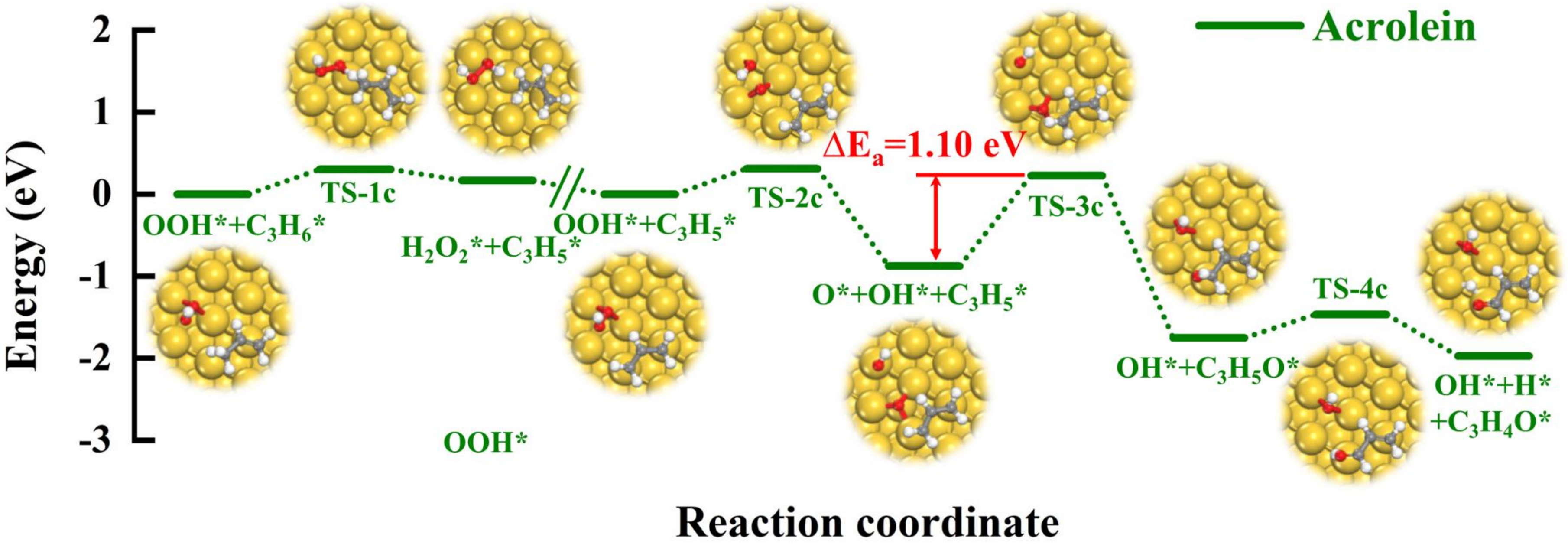

Supplementary Fig. 2. DFT calculations for acrolein formation. Calculated energy profile and the corresponding structural configurations for acrolein formation on Au(111) surface, which involves the attack of C_3H_6* by OOH* to generate C_3H_4O*, H*, and OH*.

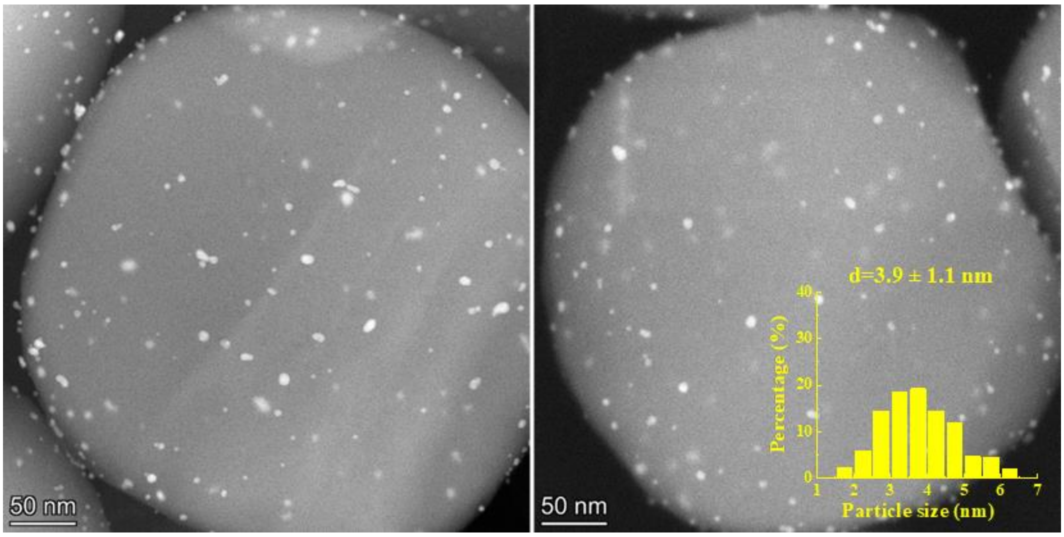

Supplementary Fig. 3. HAADF-STEM images of Au/S-1 (0.461 wt%). Typical HAADF-STEM images of Au/S-1 with the loading of 0.461 wt%.

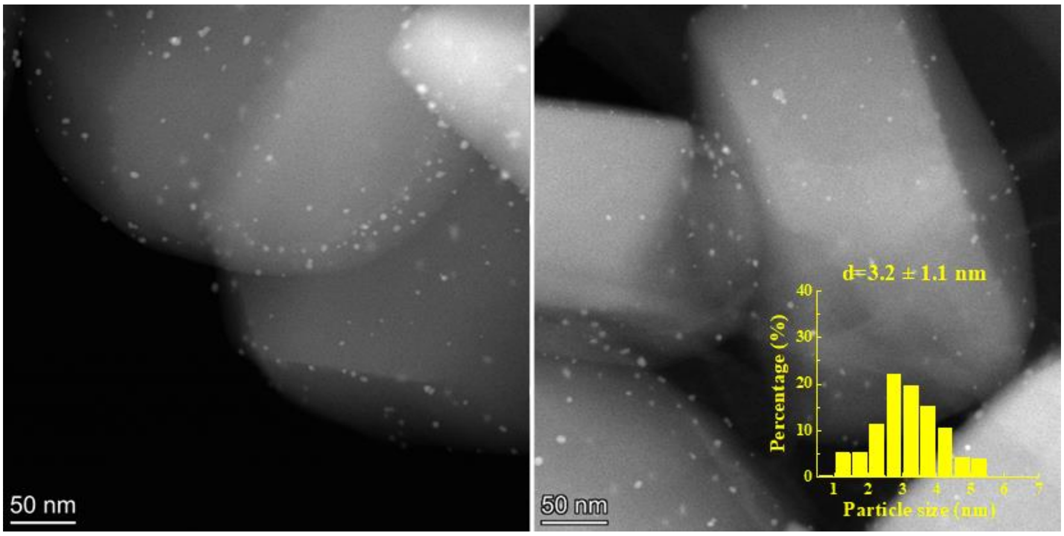

Supplementary Fig. 4. HAADF-STEM images of Au/S-1 (0.230 wt%). Typical HAADF-STEM images of Au/S-1 with the loading of 0.230 wt%.

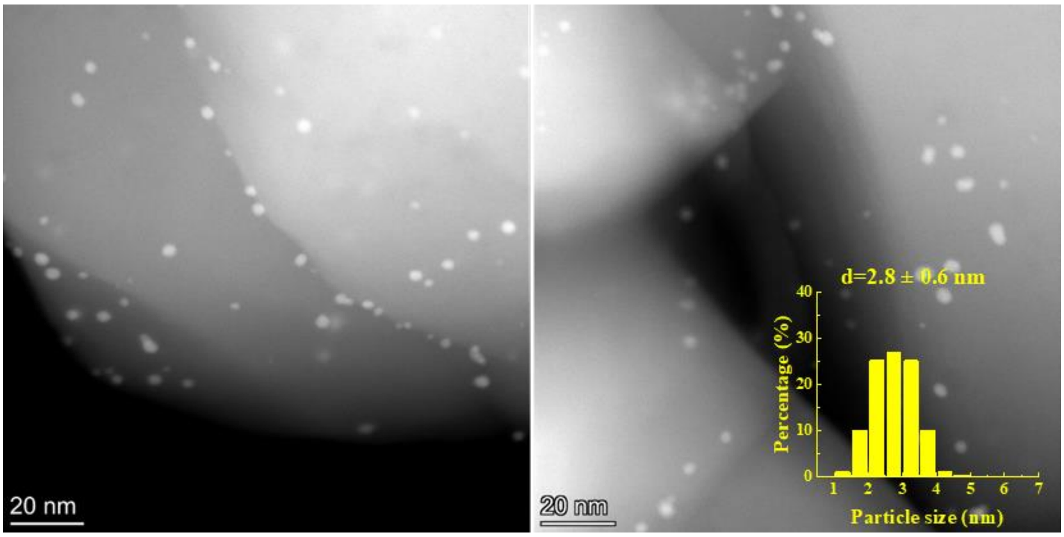

Supplementary Fig. 5. HAADF-STEM images of Au/S-1 (0.149 wt%). Typical HAADF-STEM images of Au/S-1 with the loading of 0.149 wt%.

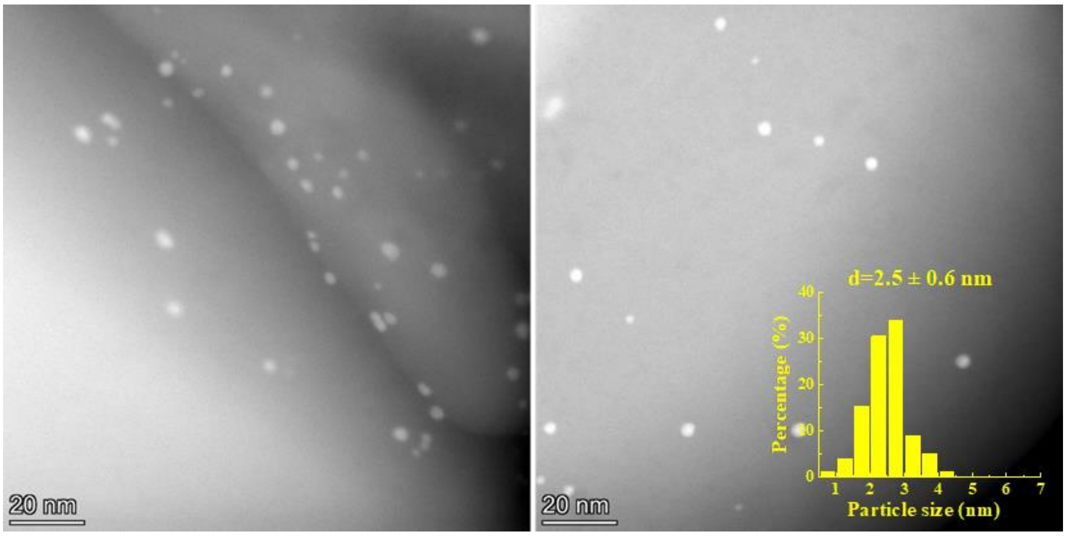

Supplementary Fig. 6. HAADF-STEM images of Au/S-1 (0.070 wt%). Typical HAADF-STEM images of Au/S-1 with the loading of 0.070 wt%.

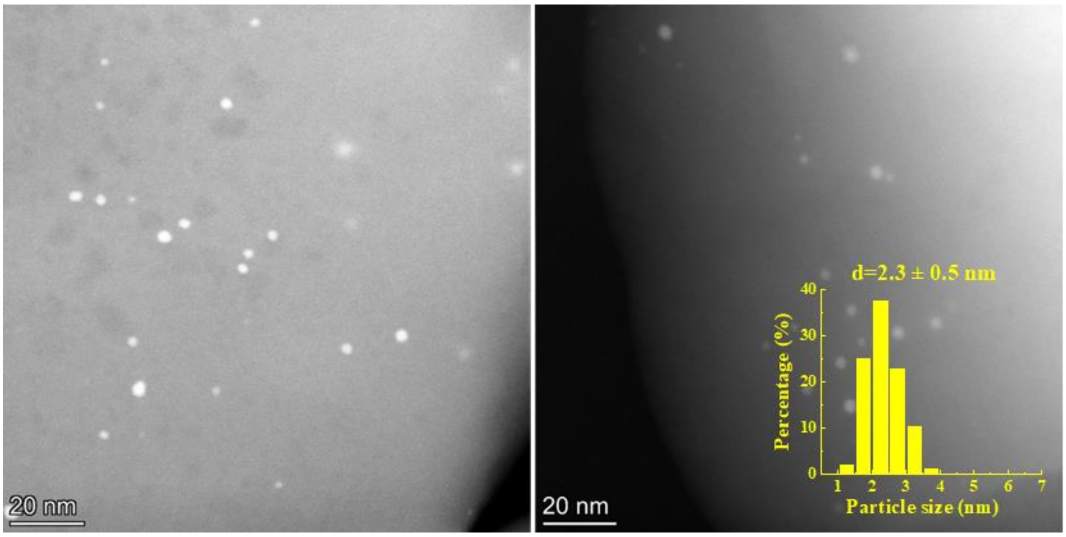

Supplementary Fig. 7. HAADF-STEM images of Au/S-1 (0.051 wt%). Typical HAADF-STEM images of Au/S-1 with the loading of 0.051 wt%.

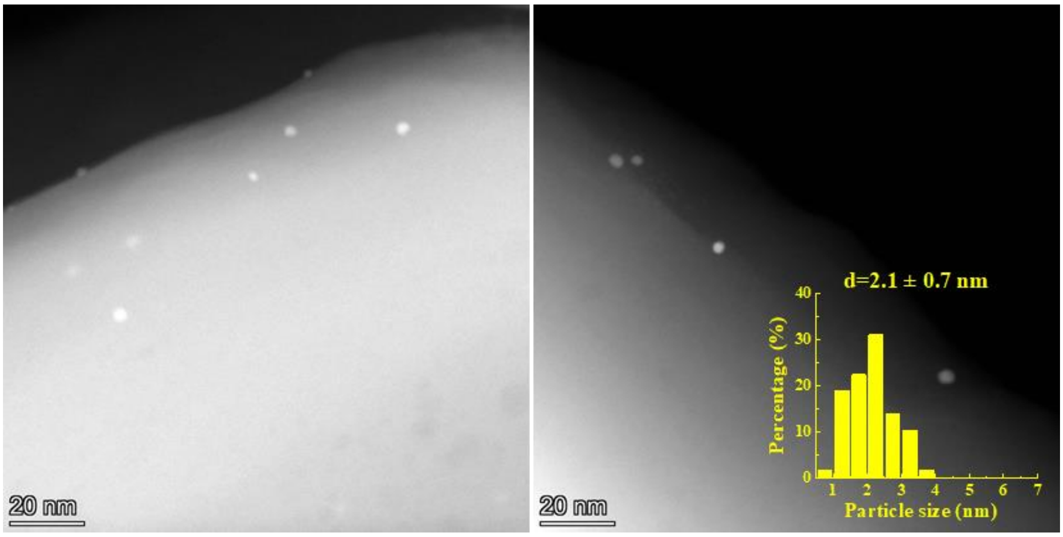

Supplementary Fig. 8. HAADF-STEM images of Au/S-1 (0.026 wt%). Typical HAADF-STEM image of Au/S-1 with the loading of 0.026 wt%.

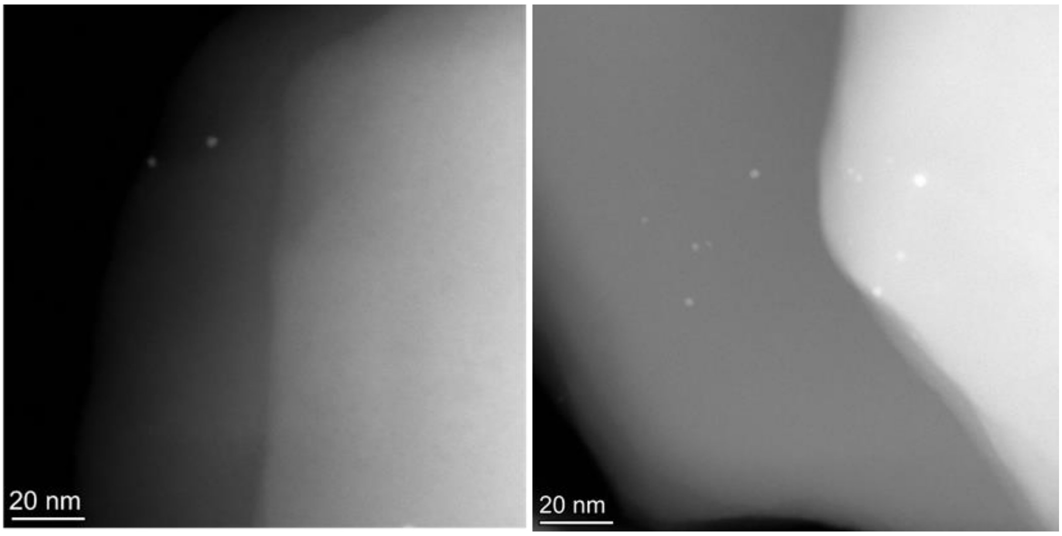

Supplementary Fig. 9. HAADF-STEM images of Au/S-1 (0.014 wt%). Typical HAADF-STEM image of Au/S-1 with the loading of 0.014 wt%.

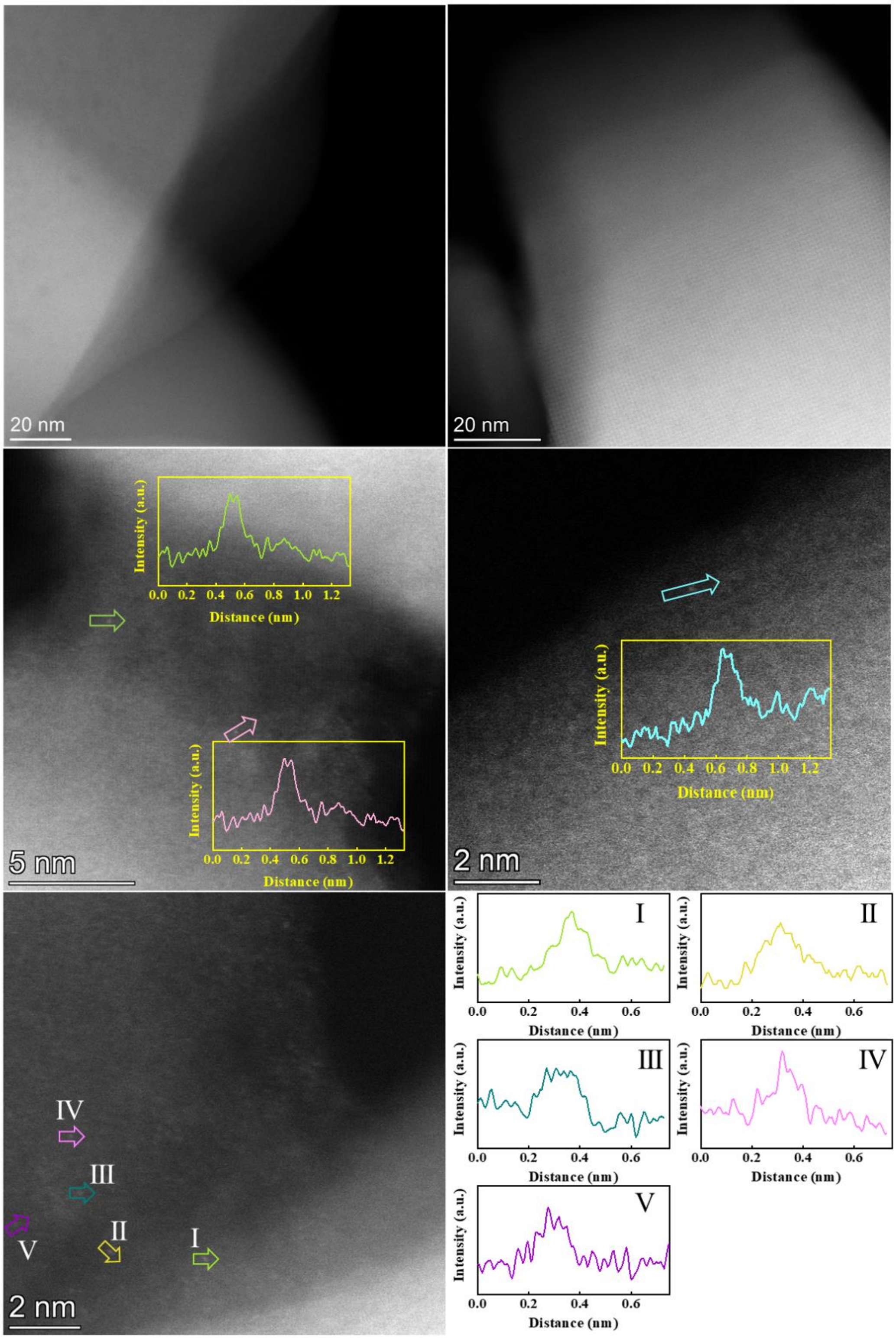

Supplementary Fig. 10. HAADF-STEM images of Au/S-1 (0.004 wt%). Typical HAADF-STEM images of Au/S-1 with the loading of 0.004 wt%.

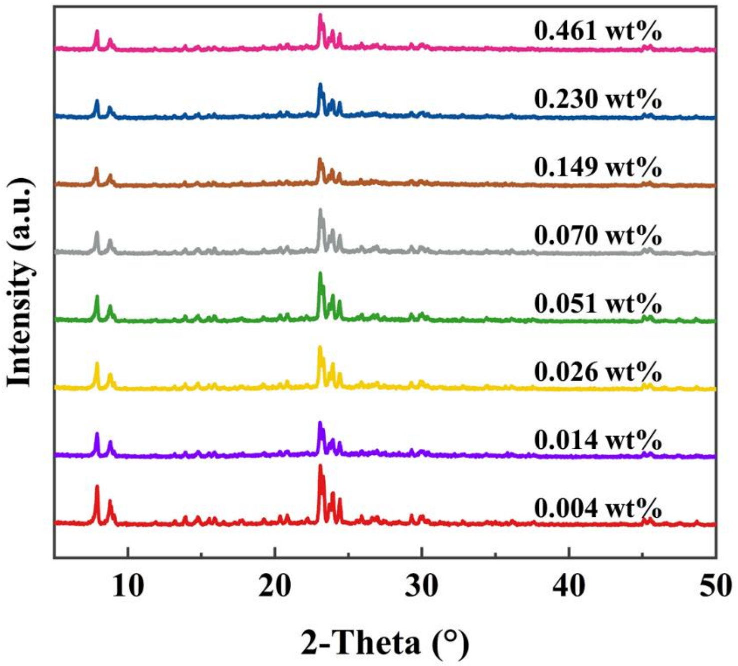

Supplementary Fig. 11. XRD patterns. XRD patterns of Au/S-1 with different loadings.

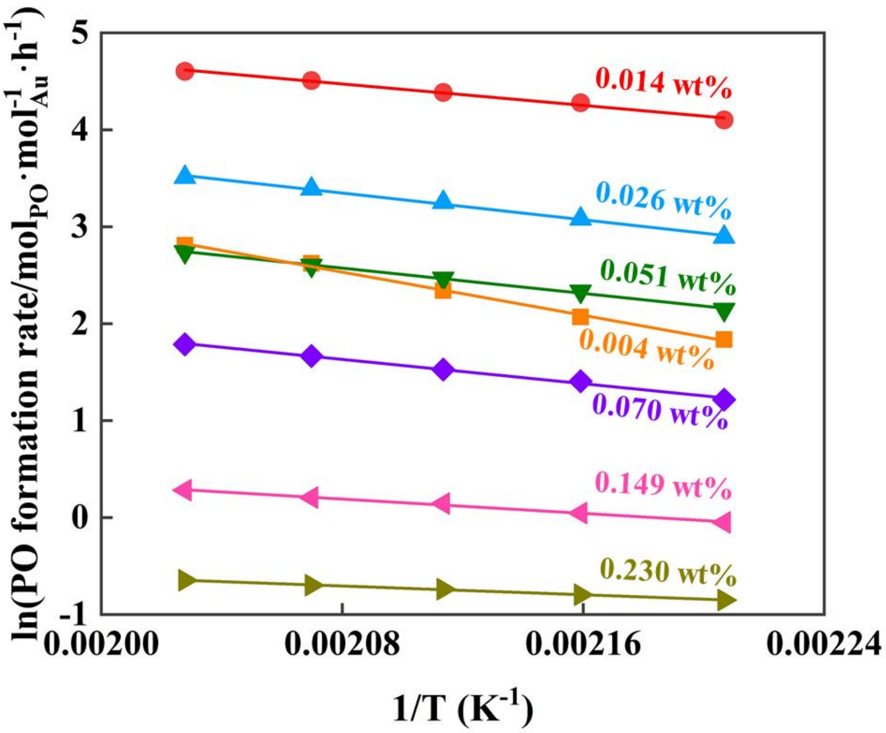

Supplementary Fig. 12. Arrhenius plots. Arrhenius plots of Au/S-1 with different loadings. Reaction conditions: 0.15 g catalyst, 35 ml·min^{-1} gas flow rate, P_{H2}:P_{O2}:P_{C3H6}:P_{N2}=1:1:1:7, 180-220 ºC.

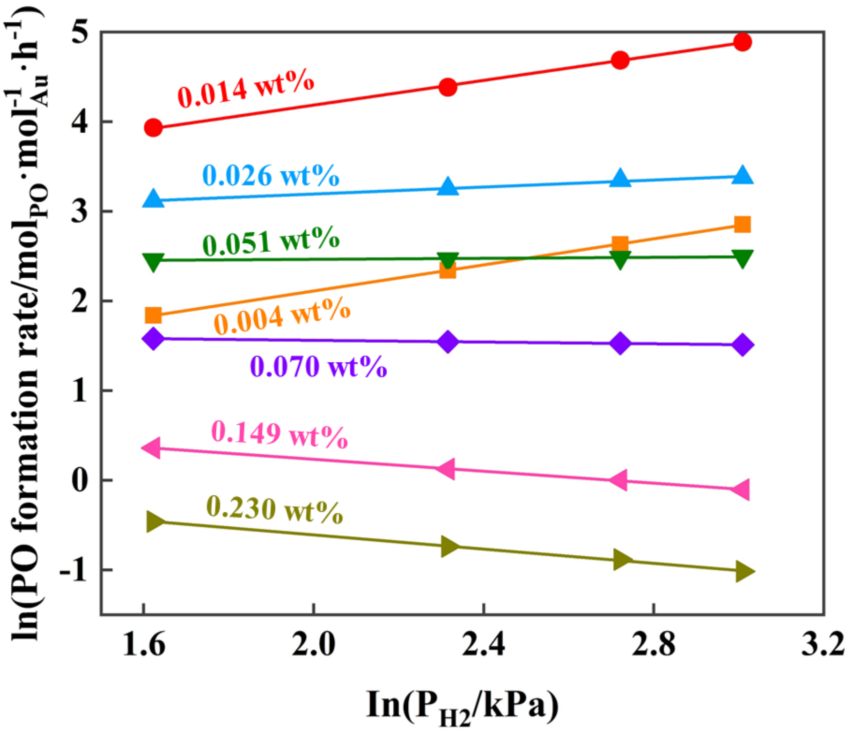

Supplementary Fig. 13. H_2 reaction order plots. Plots of the ln(PO formation rate) versus ln(P_{H2}) yield the reaction order in H_2 for Au/S-1 with different loadings. Reaction conditions: 0.15 g catalyst, 35 ml·min^{-1} gas flow rate, 200 ºC.

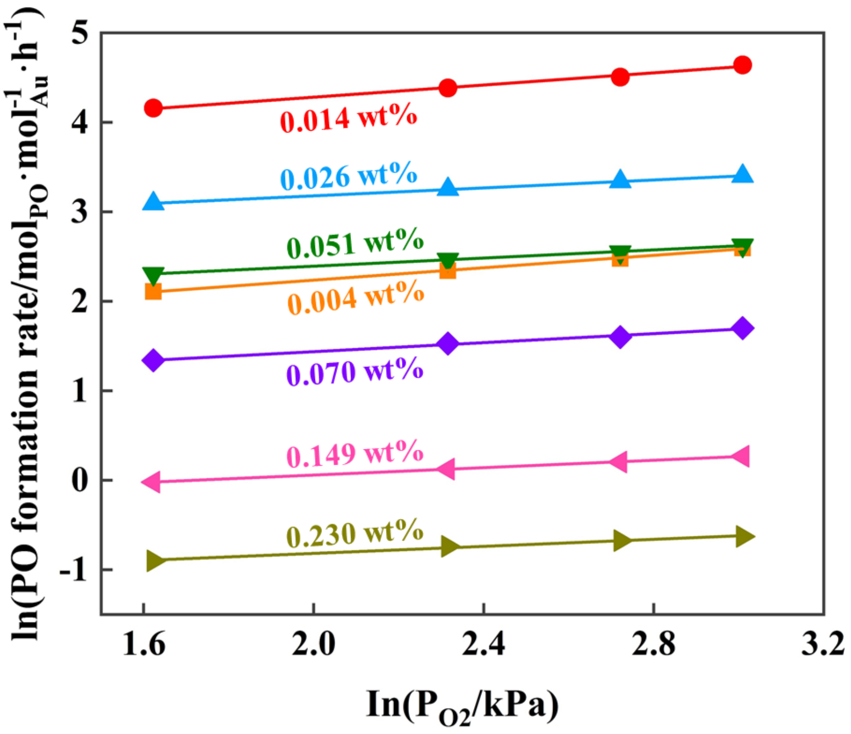

Supplementary Fig. 14. O_2 reaction order plots. Plots of the ln(PO formation rate) versus ln(P_{O2}) yield the reaction order in O_2 for Au/S-1 with different loadings. Reaction conditions: 0.15 g catalyst, 35 ml·min^{-1} gas flow rate, 200 ºC.

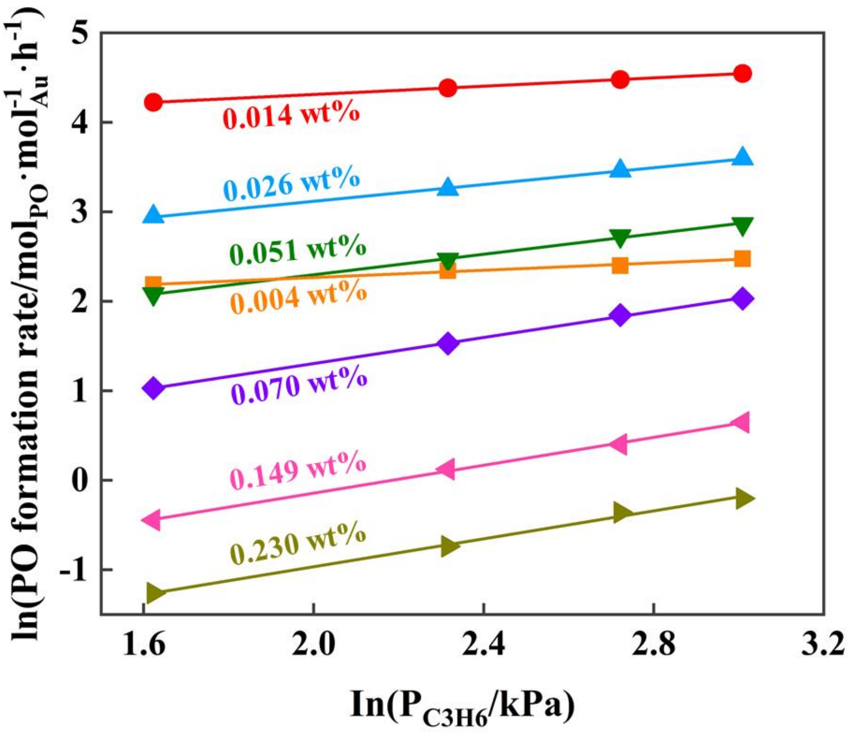

Supplementary Fig. 15. C_3H_6 reaction order plots. Plots of the ln(PO formation rate) versus ln(P_{C3H6}) yield the reaction order in C_3H_6 for Au/S-1 with different loadings. Reaction conditions: 0.15 g catalyst, 35 ml·min^{-1} gas flow rate, 200 ºC.

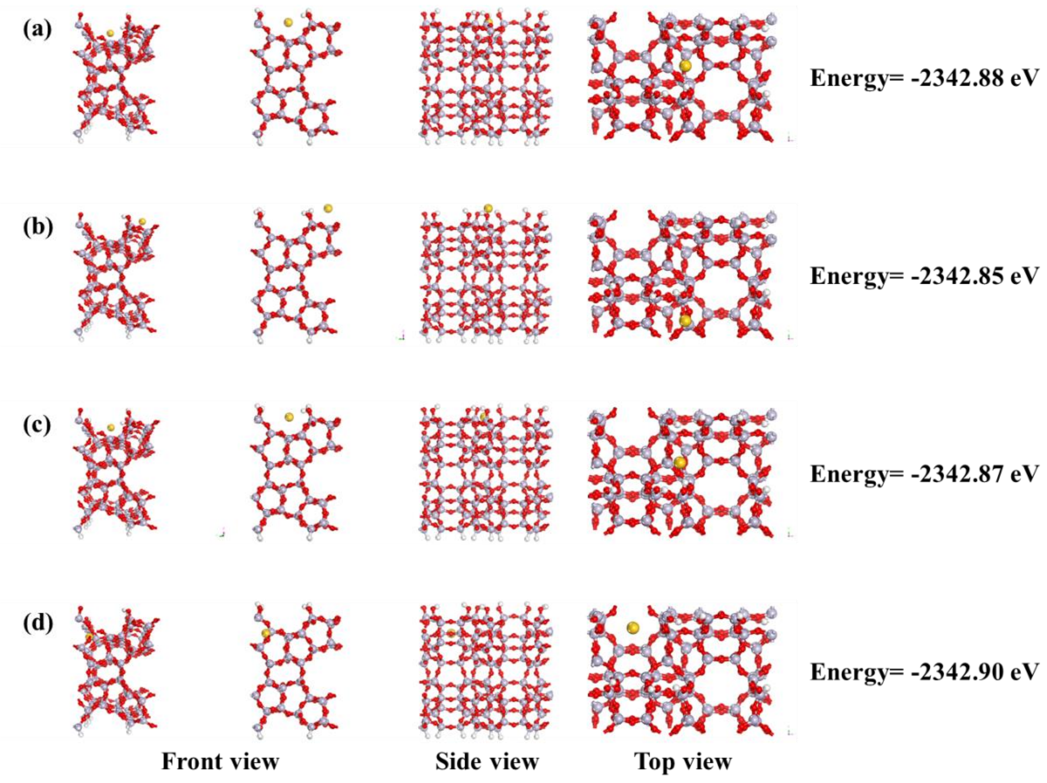

Supplementary Fig. 16. Local structure of Au single atom. Different local structure and the corresponding single point energy of Au single atom over silicalite-1.

Note: Structure (a) was selected to model the Au single atom due to its lower energy than structure (b) and structure (c). Moreover, although structure (d) has the lowest energy among all these models, the Au single atom migrates into the micropores of silicalite-1. Because the micropores are blocked by templates during silicalite-1 preparation and Au species are mainly located on its external surface, structure (d) was not selected for the DFT calculations.

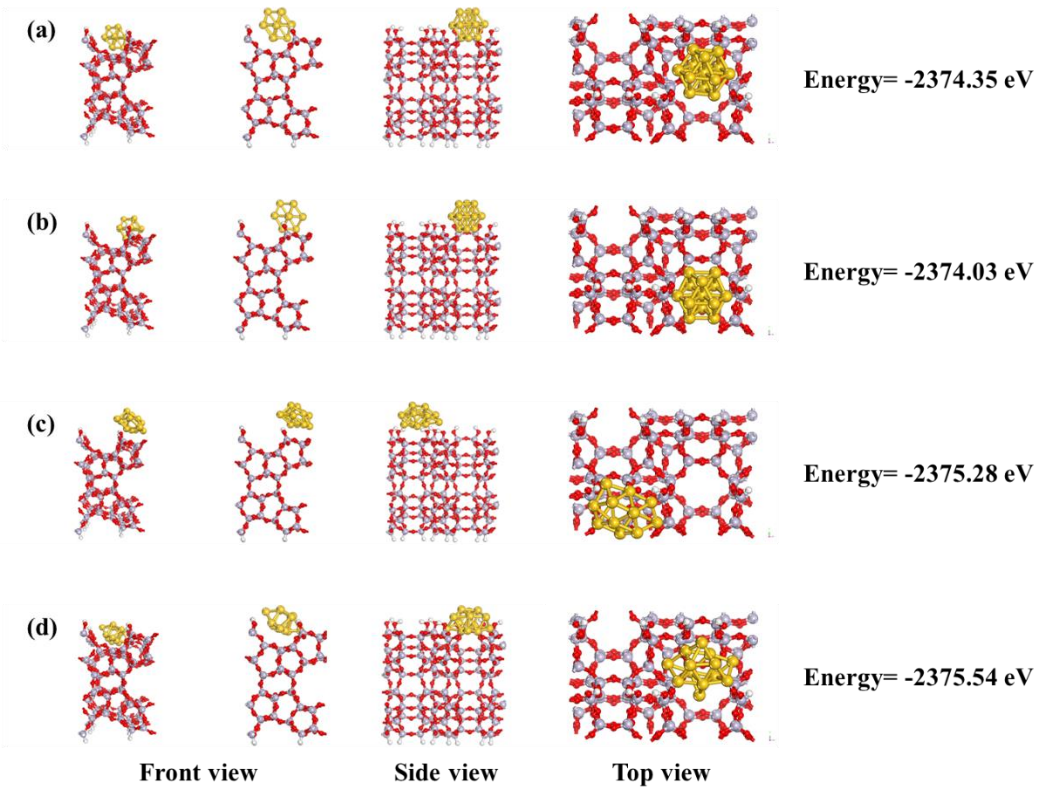

Supplementary Fig. 17. Local structure of Au_{13}. Different local structure and the corresponding single point energy of Au_{13} cluster over silicalite-1.

Note: Structure (a) was selected to model the Au_{13} cluster due to its lower energy than structure (b). Moreover, although structure (c) and structure (d) have lower than energy than structure (a), their structures exhibit a collapse after structural optimization, and thus were not selected for the DFT calculations.

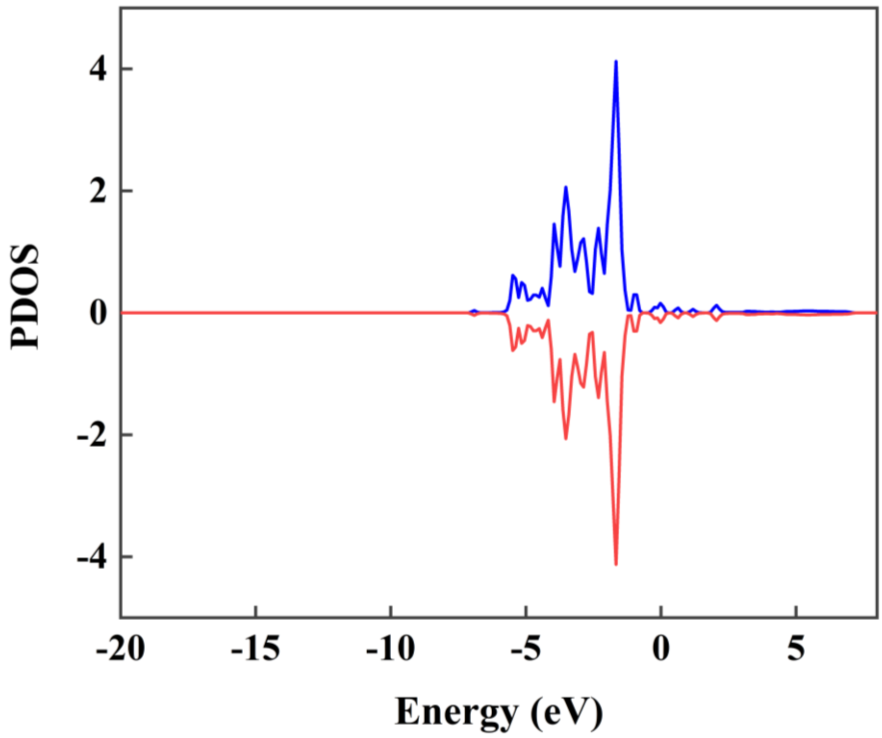

Supplementary Fig. 18. PDOS analysis. PDOS analysis of Au 5d states.

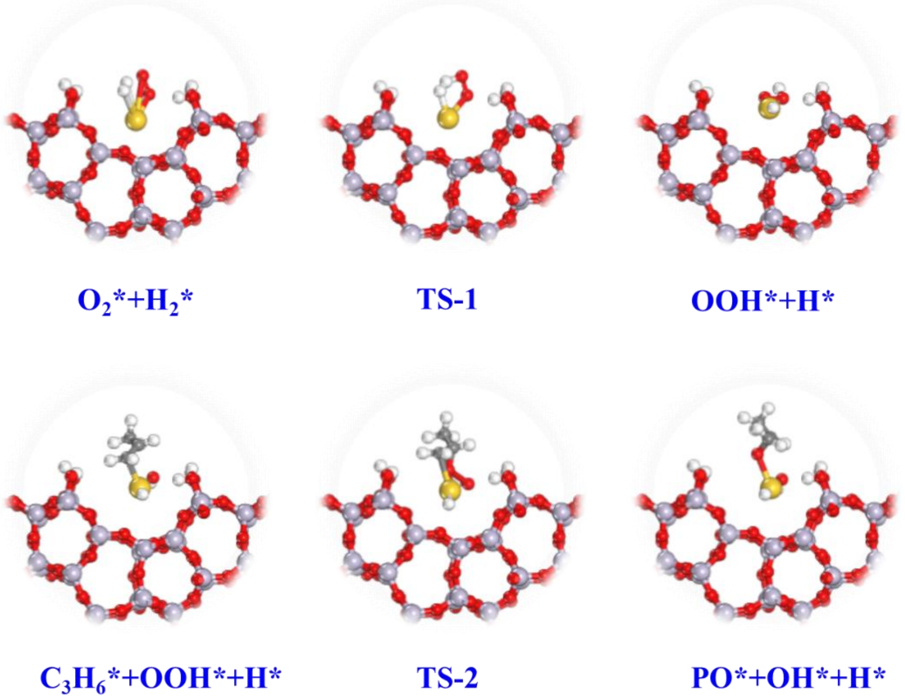

Supplementary Fig. 19. Structural configurations for Au single atom. Structural configurations for propylene epoxidation on Au single atom.

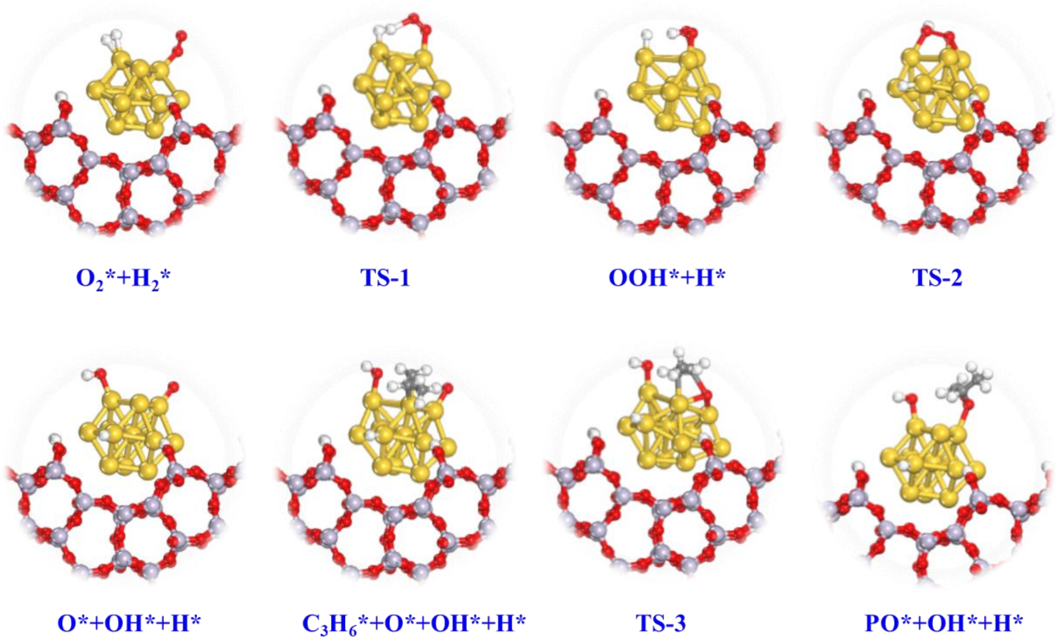

Supplementary Fig. 20. Structural configurations for Au_{13}. Structural configurations for propylene epoxidation on Au_{13} cluster.

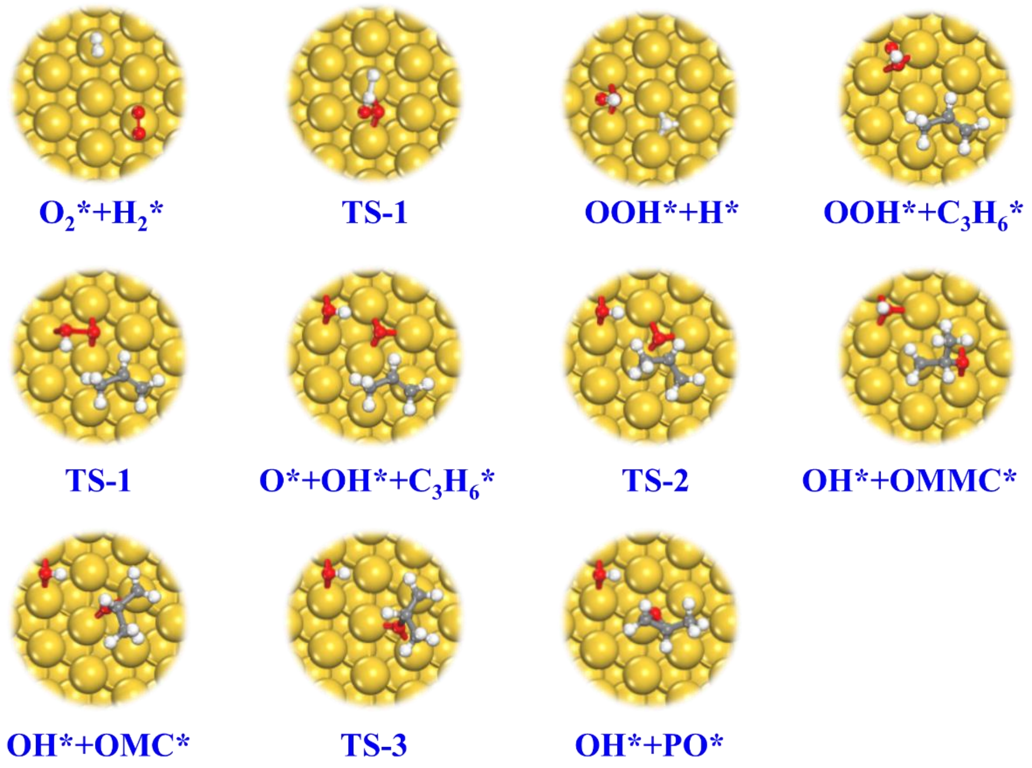

Supplementary Fig. 21. Structural configurations for Au(111). Structural configurations for propylene epoxidation on Au(111) surface.

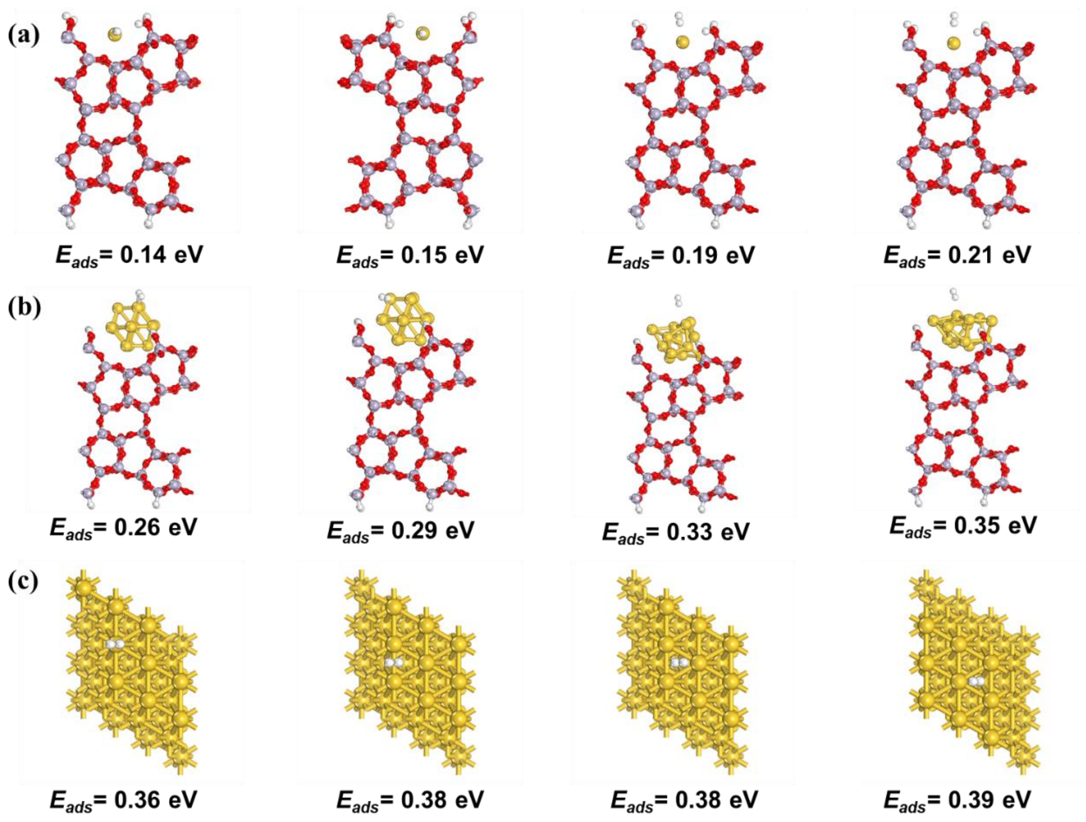

Supplementary Fig. 22. H_2 adsorption configurations. Configurations of H_2 adsorption on Au single atom (a), Au_{13} cluster (b), and Au(111) surface (c) with different binding sites, and the corresponding adsorption energies.

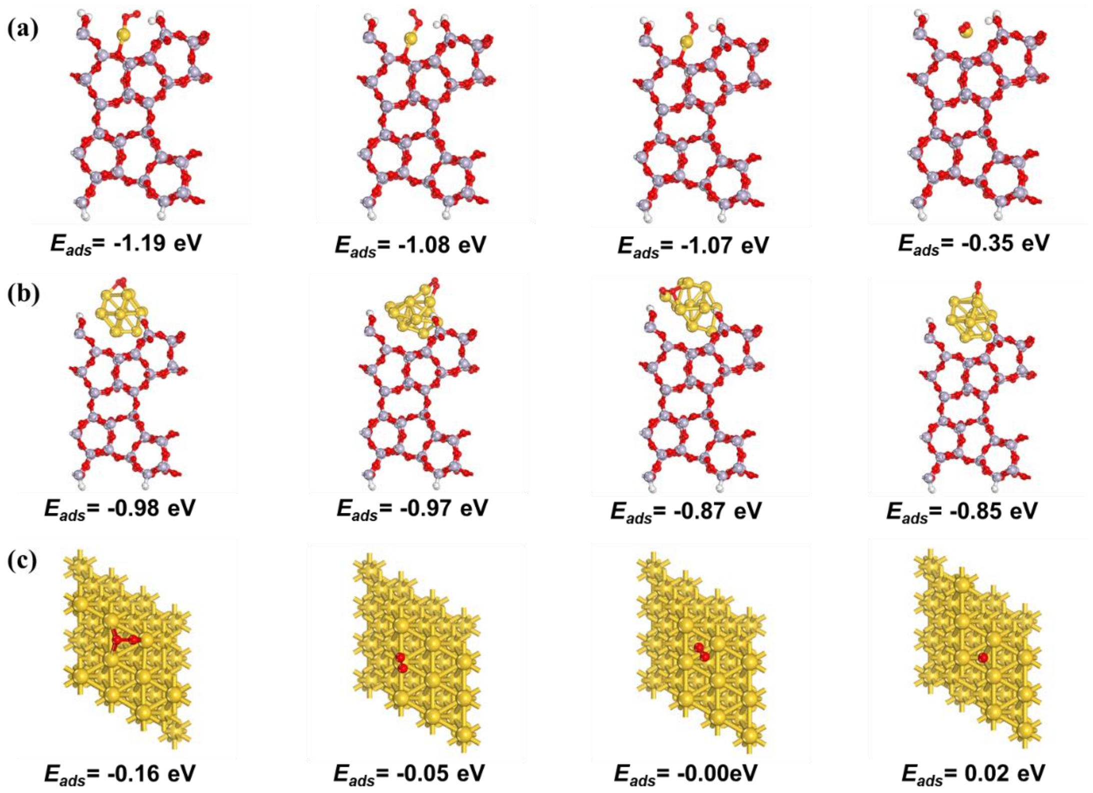

Supplementary Fig. 23. O_2 adsorption configurations. Configurations of O_2 adsorption on Au single atom (a), Au_{13} cluster (b), and Au(111) surface (c) with different binding sites, and the corresponding adsorption energies.

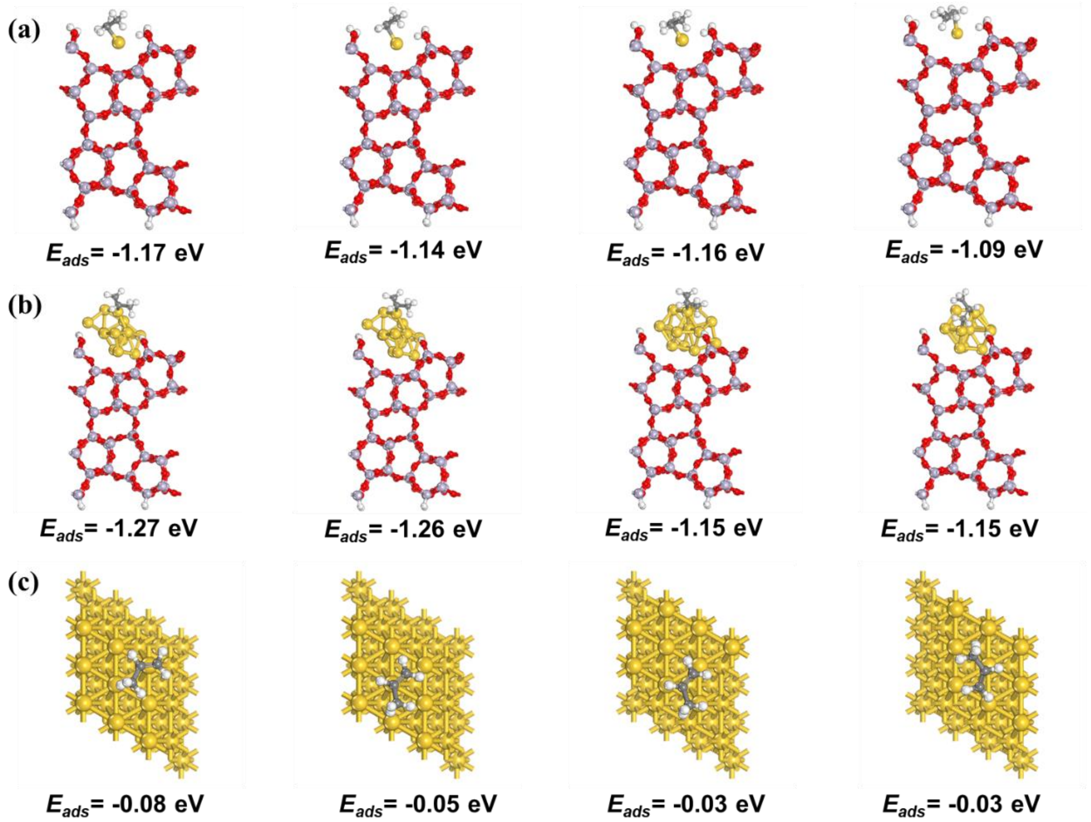

Supplementary Fig. 24. Propylene adsorption configurations. Configurations of propylene adsorption on Au single atom (a), Au_{13} cluster (b), and Au(111) surface (c) with different binding sites, and the corresponding adsorption energies.

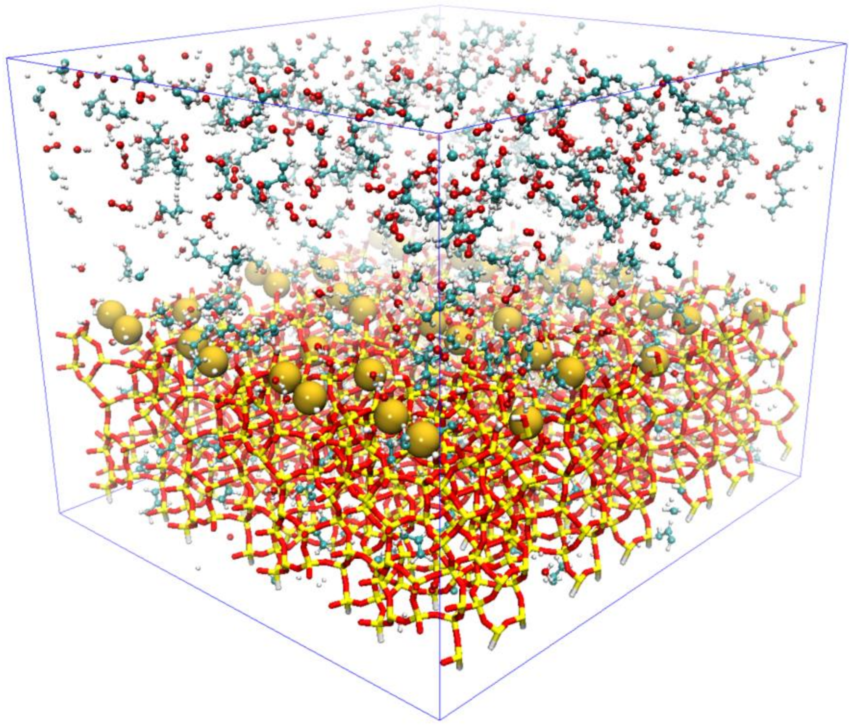

Supplementary Fig. 25. Initial snapshot for Au_1. Initial snapshot for the RMD-CMD simulations of Au_1 catalyst.

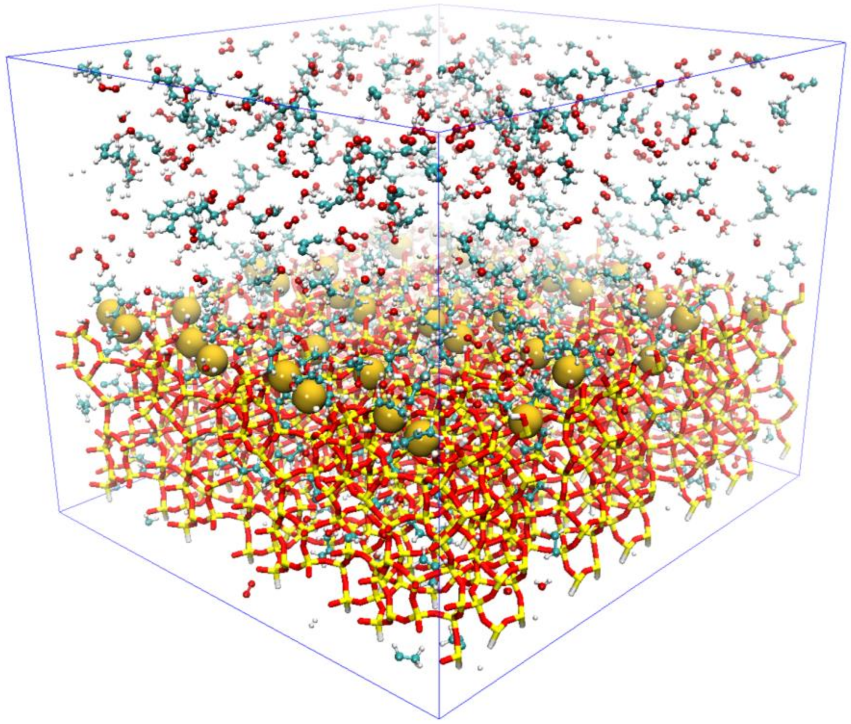

Supplementary Fig. 26. Final snapshot for Au_1. Final snapshot for the RMD-CMD simulations of Au_1 catalyst.

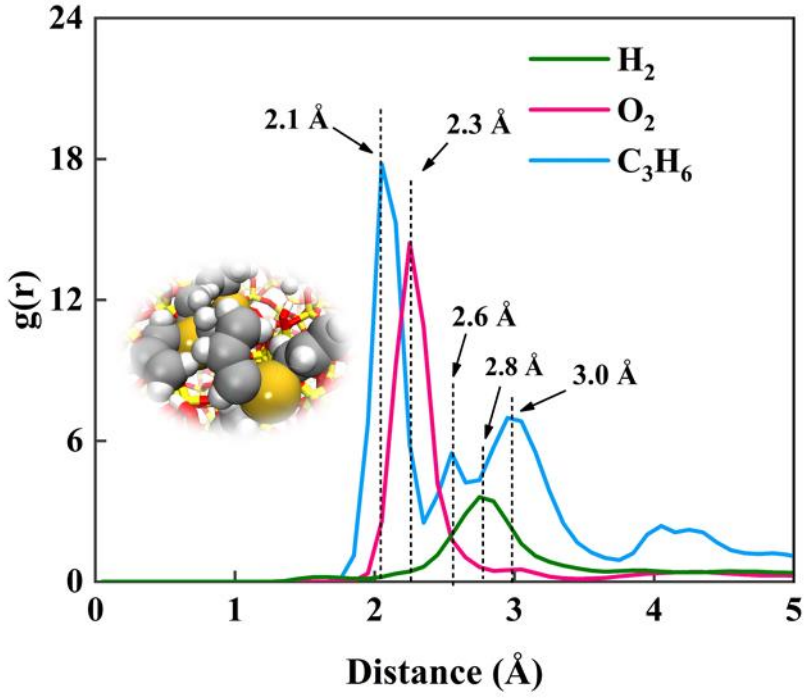

Supplementary Fig. 27. Radial distribution. Radial distribution functions (RDFs) for H_2, O_2, and propylene over Au single atoms.

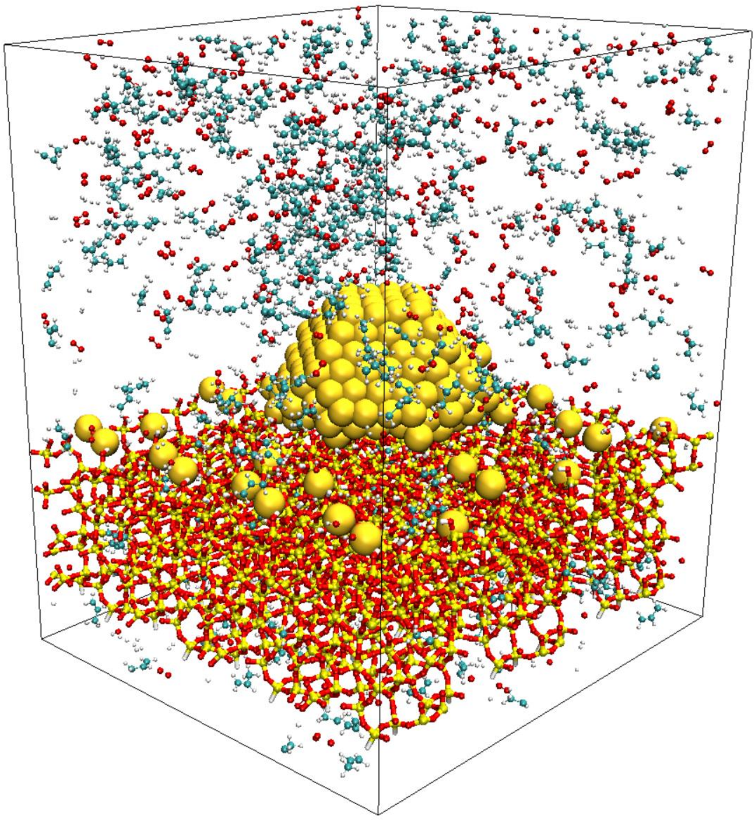

Supplementary Fig. 28. Initial snapshot for $Au_{1\&n}$. Initial snapshot for the RMD-CMD simulations of $Au_{1\&n}$ catalyst.

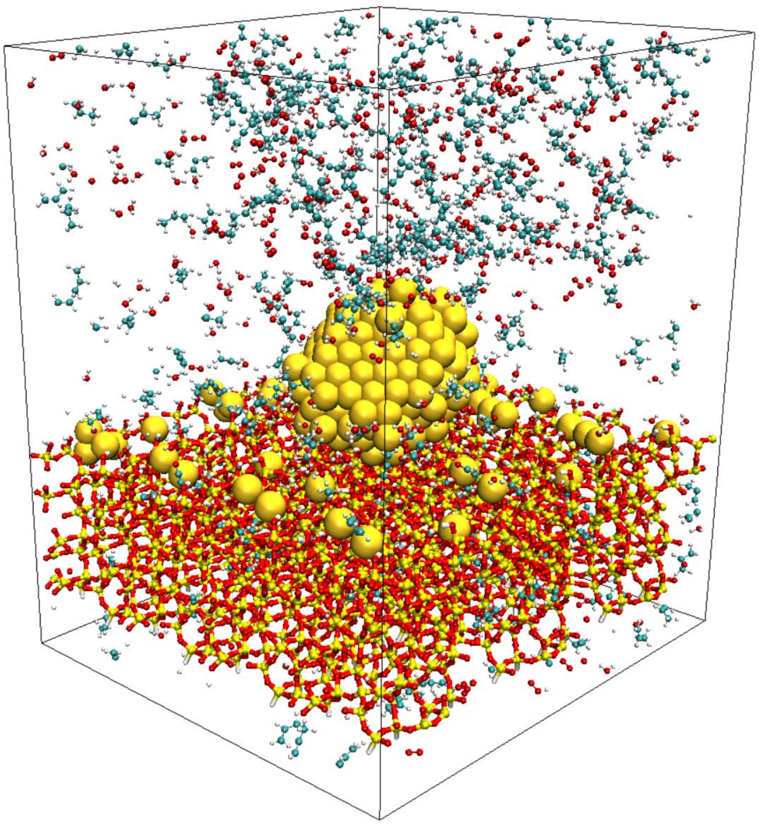

Supplementary Fig. 29. Final snapshot for $Au_{1\&n}$. Final snapshot for the RMD-CMD simulations of $Au_{1\&n}$ catalyst.

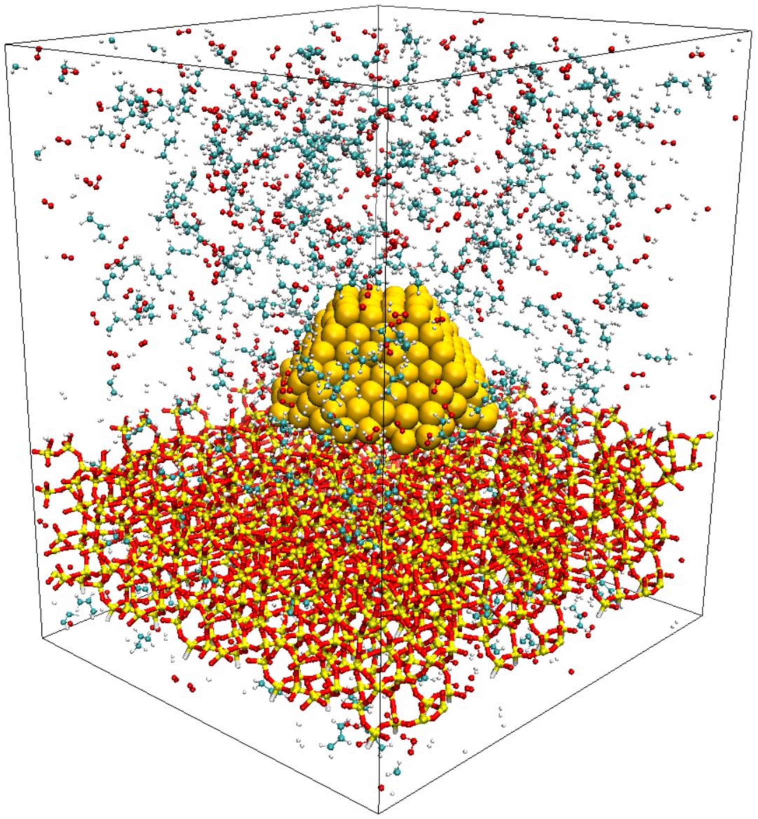

Supplementary Fig. 30. Initial snapshot for Au_n. Initial snapshot for the RMD-CMD simulations of Au_n catalyst.

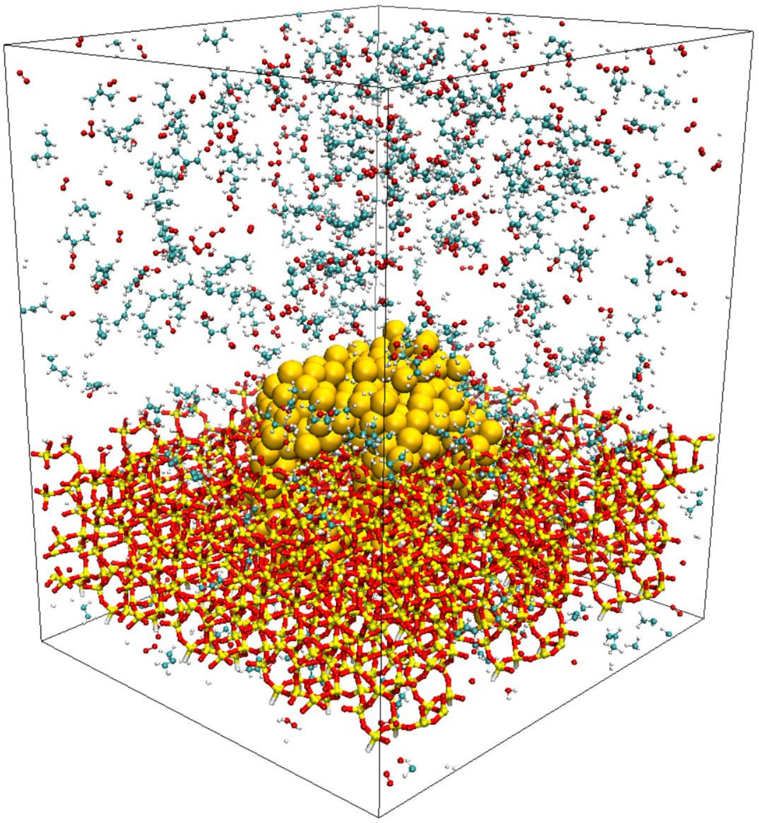

Supplementary Fig. 31. Final snapshot for Au_n. Final snapshot for the RMD-CMD simulations of Au_n catalyst.

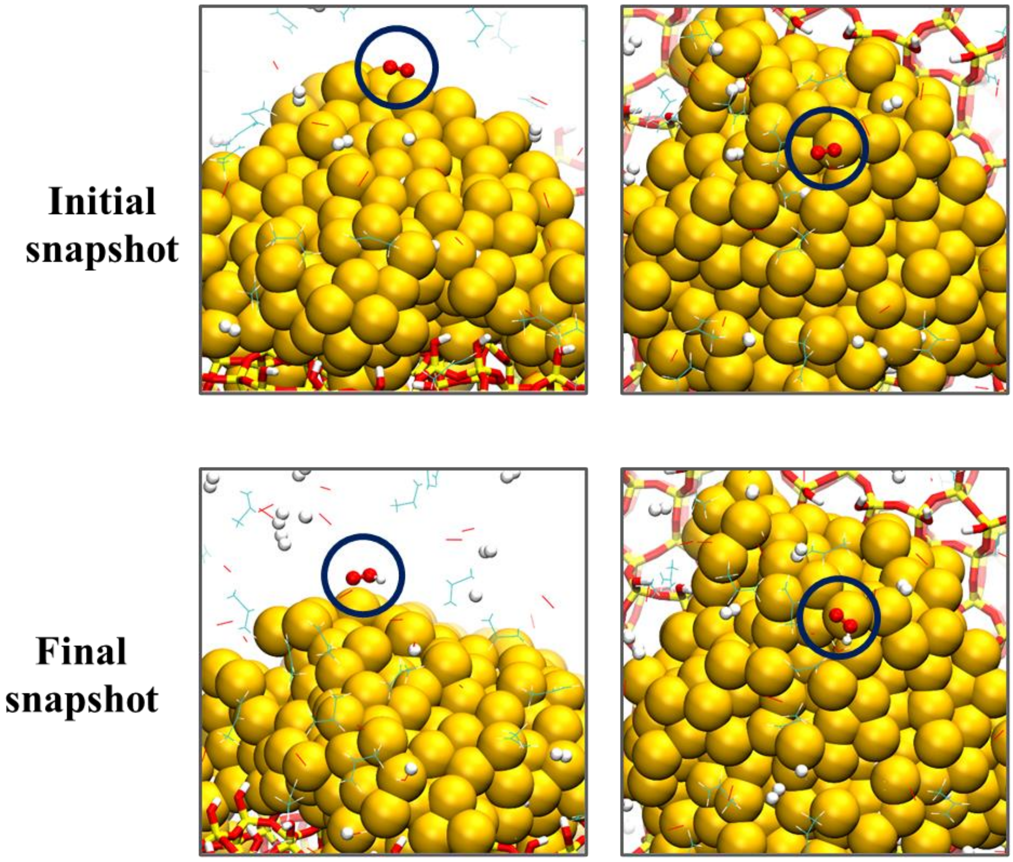

Supplementary Fig. 32. Hydroperoxyl generation. Initial and final snapshot for the generation of hydroperoxyl intermediate over Au nanoparticles.

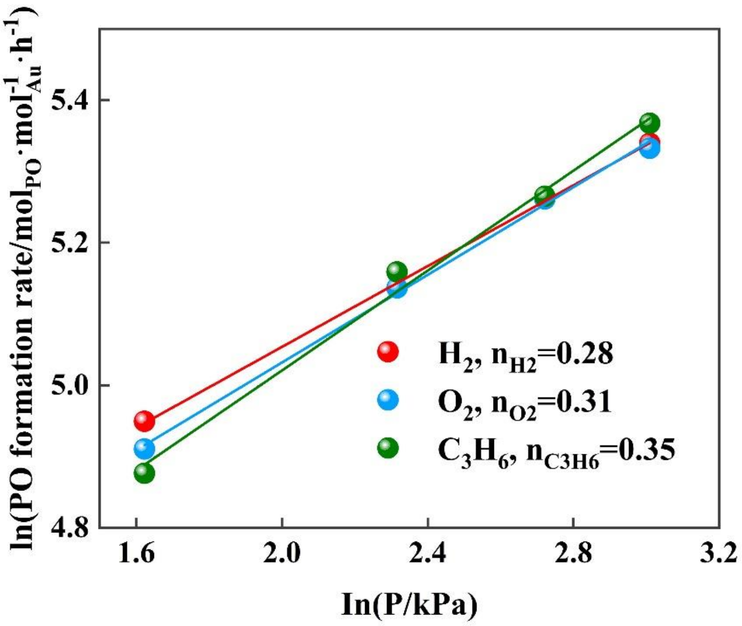

Supplementary Fig. 33. Reaction order plots for Au_n:Au_1=1:3. Plots of the ln(PO formation rate) versus ln(P) yield the reaction order in H_2, O_2, and C_3H_6 for mortar mixing of Au_n with Au_1 (Au_n:Au_1=1:3). Reaction conditions: 0.15 g catalyst, 35 ml·min^{-1} gas flow rate, 200 ºC.

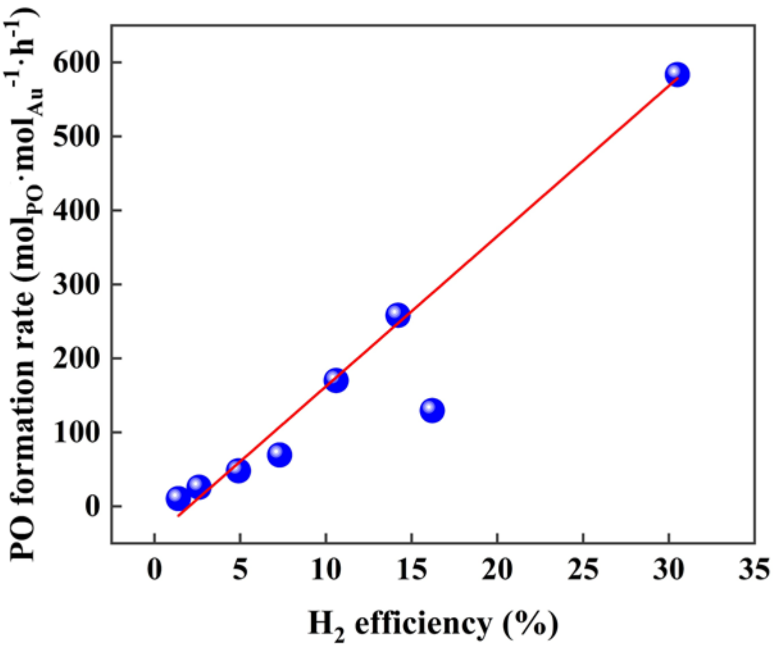

Supplementary Fig. 34. PO formation rate and H_2 efficiency correlation. The correlation between PO formation rate and H_2 efficiency for mixing Au_1 and Au_n in different compositions.

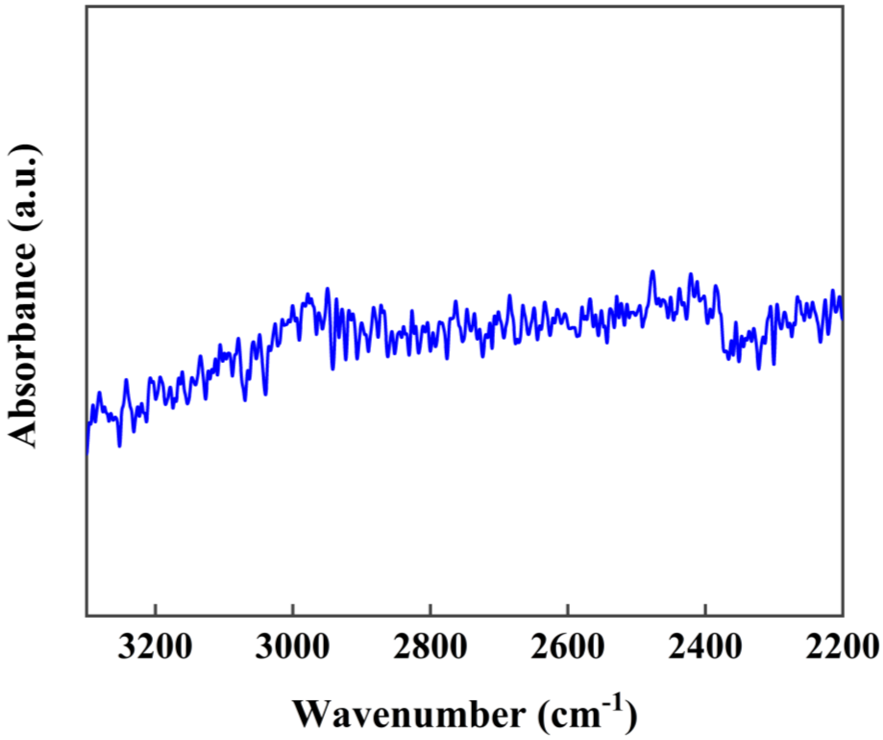

Supplementary Fig. 35. DRIFTS spectra. In-situ DRIFTS measurement of C_3H_6 adsorption over S-1 at 40 ºC.

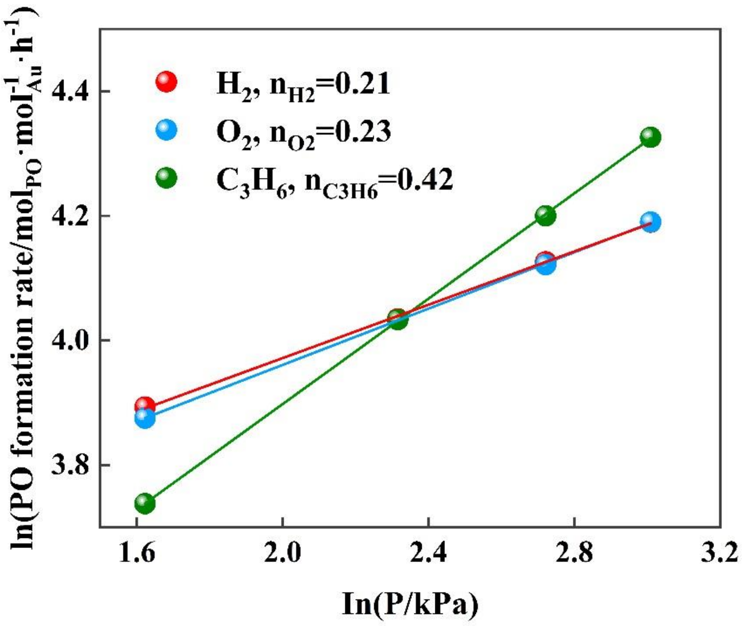

Supplementary Fig. 36. Reaction order plots for Au_n:Au_1=3:1. Plots of the ln(PO formation rate) versus ln(P) yield the reaction order in H_2, O_2, and C_3H_6 for mortar mixing of Au_n with Au_1 (Au_n:Au_1=3:1). Reaction conditions: 0.15 g catalyst, 35 ml·min^{-1} gas flow rate, 200 °C.

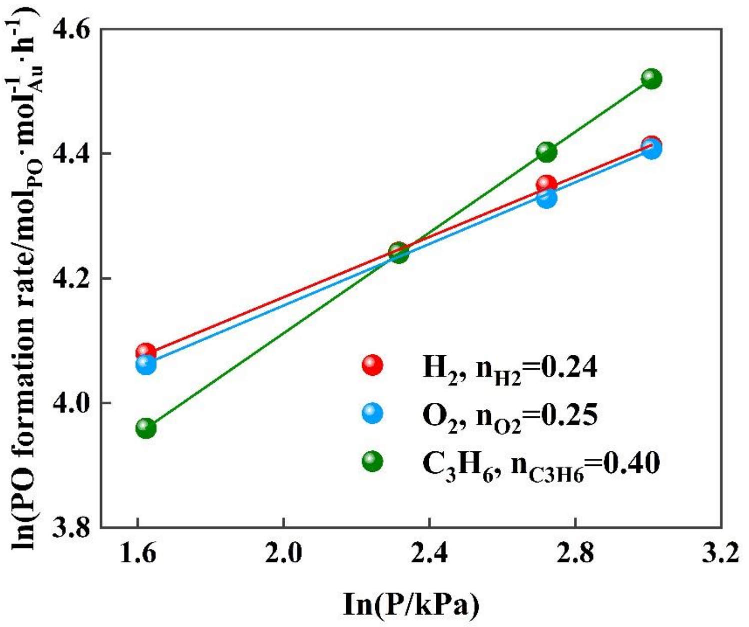

Supplementary Fig. 37. Reaction order plots for Au_n:Au_1=1:1. Plots of the ln(PO formation rate) versus ln(P) yield the reaction order in H_2, O_2, and C_3H_6 for mortar mixing of Au_n with Au_1 (Au_n:Au_1=1:1). Reaction conditions: 0.15 g catalyst, 35 ml·min^{-1} gas flow rate, 200 °C.

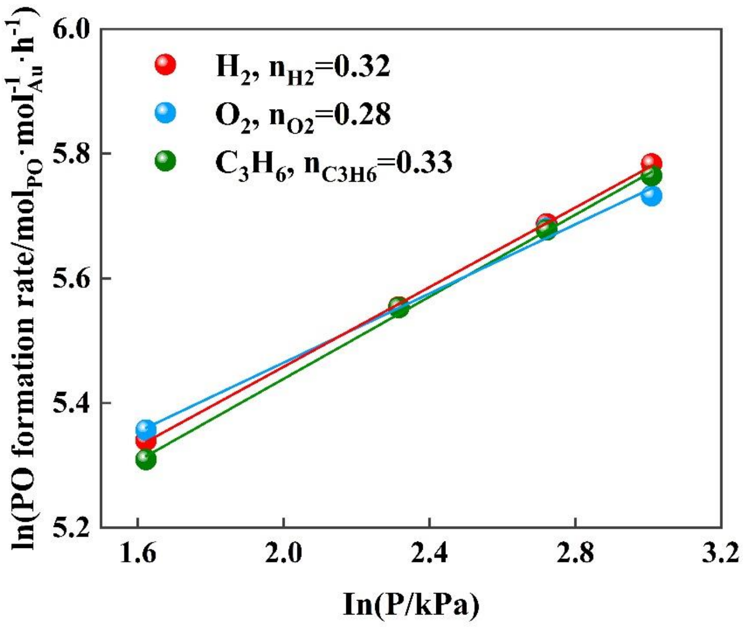

Supplementary Fig. 38. Reaction order plots for Au_n:Au_1=1:5. Plots of the ln(PO formation rate) versus ln(P) yield the reaction order in H_2, O_2, and C_3H_6 for mortar mixing of Au_n with Au_1 (Au_n:Au_1=1:5). Reaction conditions: 0.15 g catalyst, 35 ml·min^{-1} gas flow rate, 200 °C.

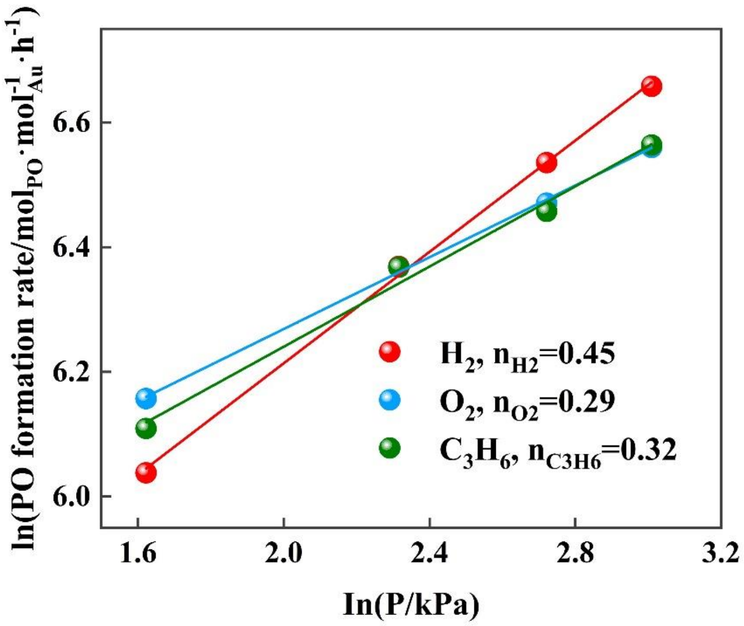

Supplementary Fig. 39. Reaction order plots for Au_n:Au_1=1:7. Plots of the ln(PO formation rate) versus ln(P) yield the reaction order in H_2, O_2, and C_3H_6 for mortar mixing of Au_n with Au_1 (Au_n:Au_1=1:7). Reaction conditions: 0.15 g catalyst, 35 ml·min^{-1} gas flow rate, 200 °C.

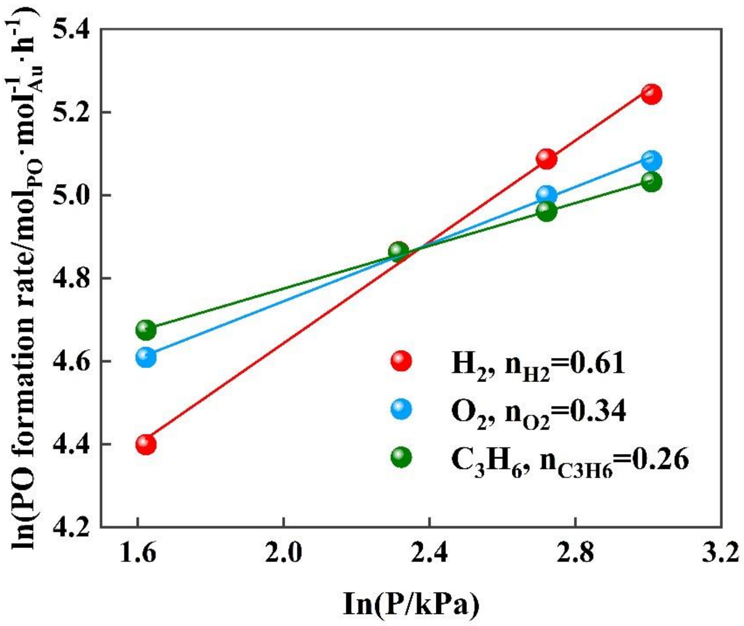

Supplementary Fig. 40. Reaction order plots for Au_n:Au_1=1:9. Plots of the ln(PO formation rate) versus ln(P) yield the reaction order in H_2, O_2, and C_3H_6 for mortar mixing of Au_n with Au_1 (Au_n:Au_1=1:9). Reaction conditions: 0.15 g catalyst, 35 ml·min^{-1} gas flow rate, 200 °C.

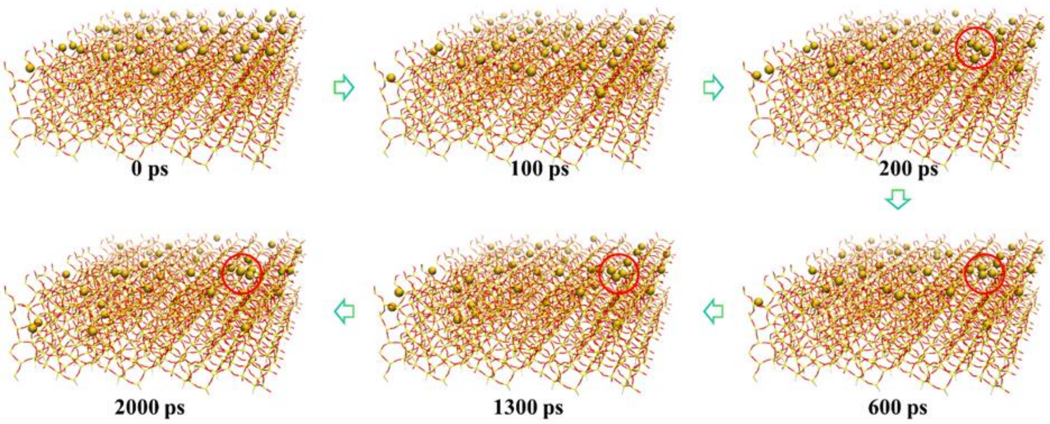

Supplementary Fig. 41. Sintering simulation. The simulation of the sintering process of the Au_1 catalyst.

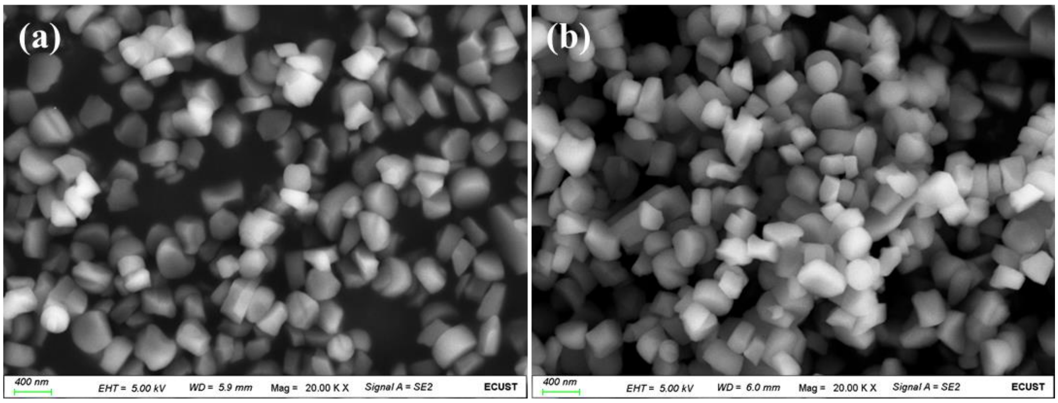

Supplementary Fig. 42. SEM images of mortar-mixing catalyst. Typical SEM images of the fresh mortar-mixing catalyst (a), and spent mortar-mixing catalyst (b).

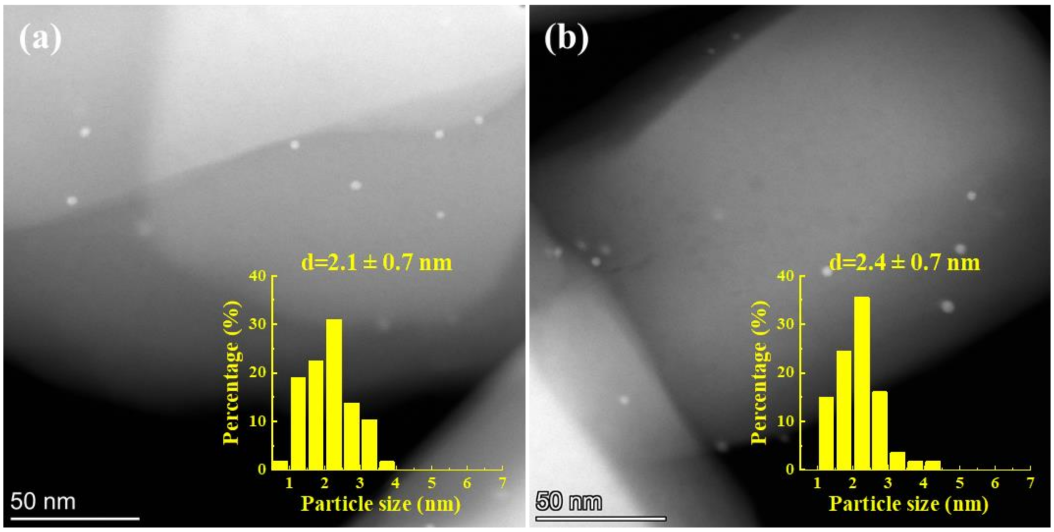

Supplementary Fig. 43. HAADF-STEM images of mortar-mixing catalyst.

Typical HAADF-STEM images of the fresh mortar-mixing catalyst (a), and spent mortar-mixing catalyst (b).

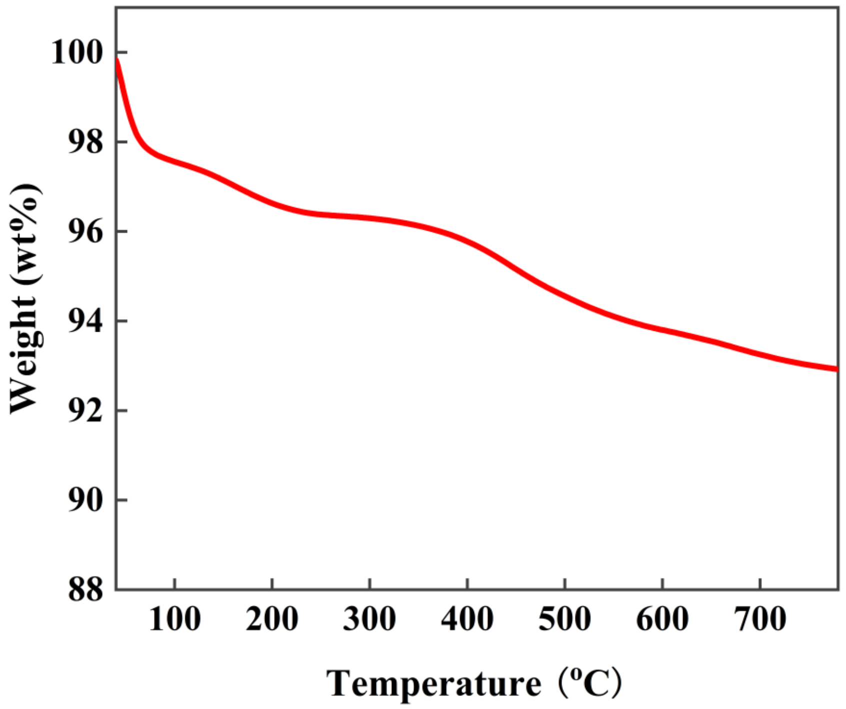

Supplementary Fig. 44. TGA analysis of mortar-mixing catalyst. TGA analysis of the spent mortar-mixing catalyst.

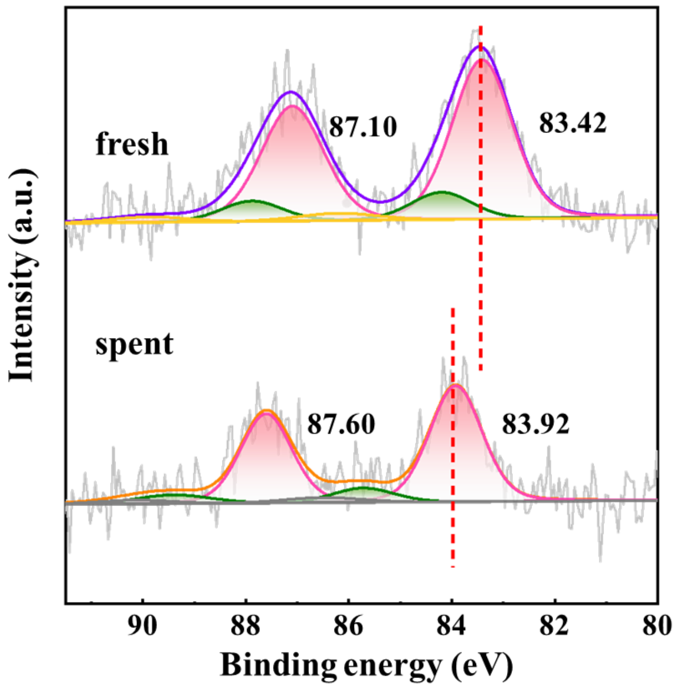

Supplementary Fig. 45. XPS spectra of mortar-mixing catalyst. XPS Au 4*f* spectra of the fresh and spent mortar-mixing catalyst. The decrease in signal for the spent catalyst indicate the poisoning by carbonaceous deposits.

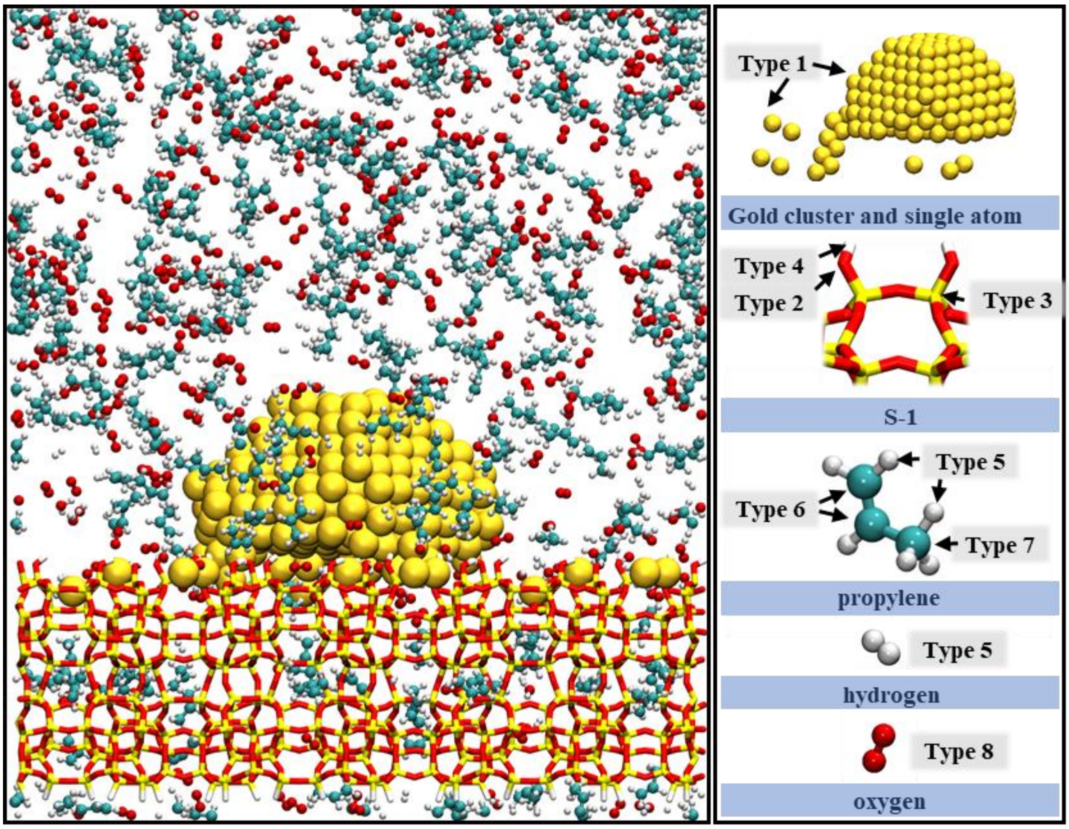

Supplementary Fig. 46. Simulation configuration. Simulation configuration and atomic type assignment.

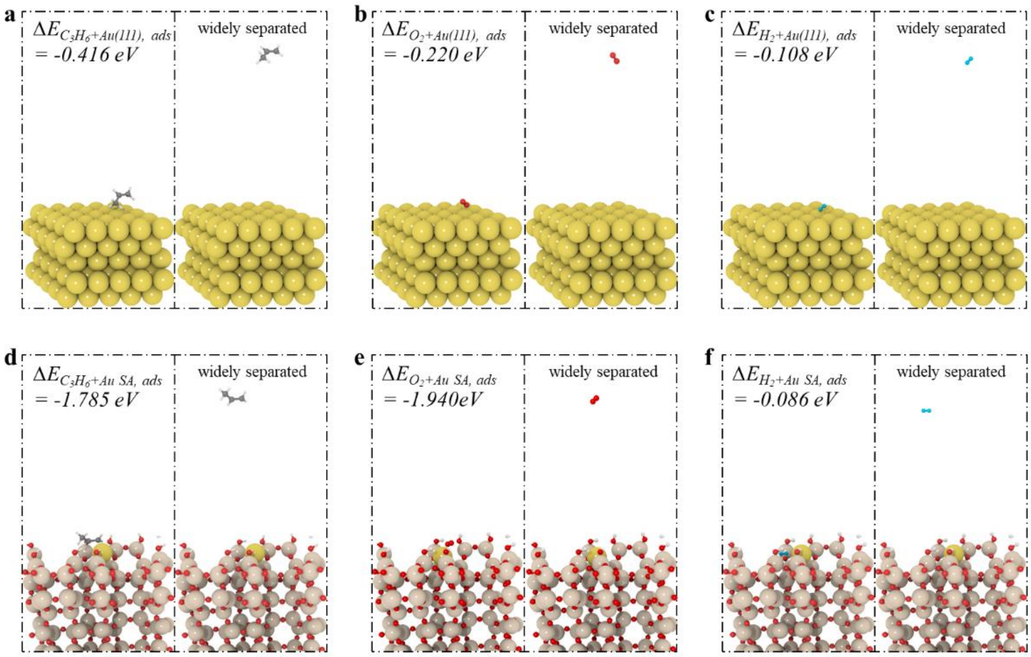

Supplementary Fig. 47. Adsorption configurations of Au(111) and Au single atom. The adsorption configurations and corresponding energies (ΔE_{ads}) of C_3H_6 (**a**), O_2 (**b**), and H_2 (**c**) on Au(111), as well as C_3H_6 (**d**), O_2 (**e**), and H_2 (**f**) on Au single atom.

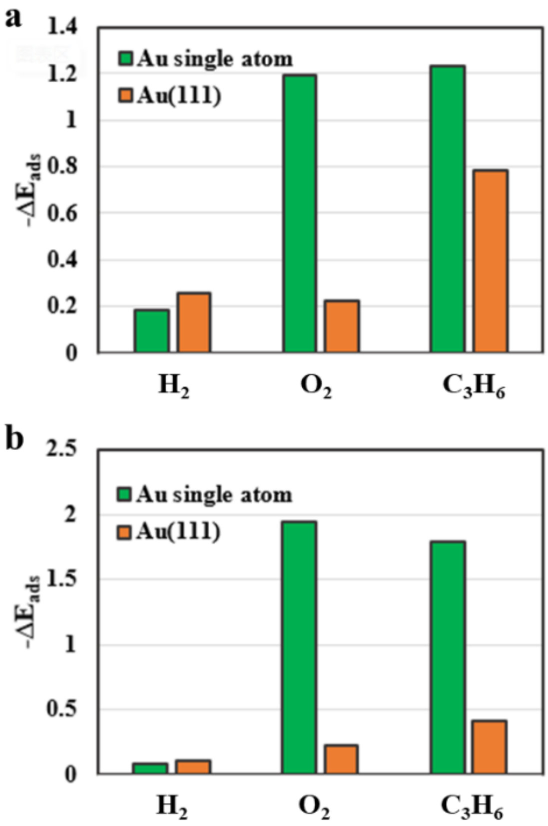

Supplementary Fig. 48. Adsorption energy comparison. The comparison of the adsorption energy between DFT calculations (**a**) and ReaxFF simulations (**b**).

Supplementary References

1. Baerlocher, C., McCusker, J. K. Database of Zeolite Structures: http://www.iza-structure.org/databases/.
2. Wu, E. L., Lawton, S. L., Olson, D. H., Rohrman, A. C. & Kokotailo, G. T. ZSM-5-type materials. Factors affecting crystal symmetry. *J. Phys. Chem.* **83**, 2777-2781 (1979).
3. Olson, D. H., Kokotailo, G. T., Lawton, S. L. & Meier, W. M. Crystal structure and structure-related properties of ZSM-5. *J. Phys. Chem.* **85**, 2238-2243 (1981).
4. Treps, L., Gomez, A., de Bruin, T. & Chizallet, C. Environment, stability and acidity of external surface sites of silicalite-1 and ZSM-5 micro and nano slabs, sheets, and crystals. *ACS Catal.* **10**, 3297-3312 (2020).
5. Hoover, W. G. Canonical dynamics: Equilibrium phase-space distributions. *Phys. Rev. A* **31**, 1695 (1985).
6. Feng, X., Duan, X., Qian, G., Zhou, X., Chen, D. & Yuan, W. Insights into size-dependent activity and active sites of Au nanoparticles supported on TS-1 for propene epoxidation with H_2 and O_2. *J. Catal.* **317**, 99-104 (2014).
