# Supplementary material for: Nanoparticles as an antidote for poisoned gold single-atom catalysts in sustainable propylene epoxidation
Source: Nat Commun. 2024 Apr 16;15:3249. doi: 10.1038/s41467-024-47538-4 (PMC11021464; doi:10.1038/s41467-024-47538-4)
Supplement: Supplementary file 3 — Description of Additional Supplementary Files [file 41467_2024_47538_MOESM3_ESM.pdf]

Description of Additional Supplementary Files

File Name: Supplementary Movie 1
Description: Video of propylene epoxidation over Au single atoms.

File Name: Supplementary Movie 2
Description: Video of propylene epoxidation over Au nanoparticle.
